# Topical trichloroacetic acid versus electrocautery for treatment of anal intraepithelial neoplasia in people living with HIV: a multicentre, randomised, non-inferiority trial (TECAIN-study)

**DOI:** 10.1007/s15010-026-02882-z

**Published:** 2026-07-10

**Authors:** Stefan Esser, Alexander Kreuter, Anja Potthoff, Robert Jablonka, Mark Oette, Martin F. Sprinzl, Daniel Exner, Konstantinos Bilbilis, Hildegard Lax, Eva-Maria Huessler, Steffi Silling, Ulrike Wieland

**Affiliations:** 1 Department of Dermatology and Venereology, Clinic for Dermatology and Venerology, University Hospital Essen, University Duisburg-Essen, Hufelandstr. 55, 45122 Essen, Germany; 2 HPSTD (HIV/AIDS, Proctology, STD) Outpatient Clinic, University Hospital Essen, University Duisburg-Essen, Essen, Germany; 3 Department of Dermatology, Venereology and Allergology, Helios St. Elisabeth Hospital, University Witten/Herdecke, Oberhausen, Germany; 4 WIR – Walk In RuhrInterdisciplinary Immunological Outpatient Clinic, Center for Sexual Health and Medicine, Department of Dermatology, Venereology and Allergology, Ruhr University Bochum, Bochum, Germany; 5 Present Address: Derma Center, Rietkötter & Jablonka, Gelsenkirchen, Germany; 6 Department of Infectious Diseases, Augustinerinnen Hospital, Cologne, Germany; 7 Department of Internal Medicine I, University Medical Center of the Johannes Gutenberg University Mainz, Mainz, Germany; 8 Department of General, Visceral, Thoracic and Vascular Surgery, Colorectal Surgery and Proctology, University Hospital Bonn, Bonn, Germany; 9 Present Address: Department of General, Visceral and Coloproctological Surgery, Evangelical Amalie Sieveking Hospital, Hamburg, Germany; 10 Centre for Clinical Studies Essen, c/o IMIBE, University Hospital Essen, Essen, Germany; 11 Institute for Medical Informatics, Biometry and Epidemiology (IMIBE), University Hospital Essen, Essen, Germany; 12 Institute of Virology, National Reference Centre for Papilloma-and Polyomaviruses, Faculty of Medicine, University of Cologne, University Hospital Cologne, Cologne, Germany

**Keywords:** Anal intraepithelial neoplasia (AIN), Anal high-grade squamous intraepithelial lesion (HSIL), HIV, Trichloroacetic acid (TCA), Electrocautery, Randomised controlled trial (RCT)

## Abstract

**Purpose:**

To compare efficacy and safety of topical trichloroacetic acid (TCA) versus electrocautery (ECA) for the treatment of human papillomavirus (HPV)-associated anal intraepithelial neoplasia (AIN) in people living with HIV (PLWH).

**Methods:**

The TECAIN-study was a prospective, multicentre, randomised (1:1 block-randomisation), open-label, non-inferiority trial in PLWH with histologically confirmed AIN recruited from seven German proctological units. The primary endpoint (PE) was therapeutic success defined as a combination of complete clinical response evaluated by high-resolution-anoscopy and histological AIN-clearance/regression four weeks after the end-of-treatment (4-weeks-follow-up, 4WFU). Secondary endpoints were assessed at 4WFU and 24WFU. Efficacy was analysed in the intention-to-treat-population as primary analysis with a non-inferiority margin of -12%.

**Results:**

Of 659 PLWH screened with HRA, 257 patients with AIN were randomised. Therapeutic outcome was assessed in 233 PLWH (TCA *n* = 118, ECA *n* = 115). The PE was reached in 62 patients of the TCA-group (52.5%) versus 71 patients of the ECA-group (61.7%) (difference in proportion − 9.2%, 95%CI -21.8% to 3.5%). While non-inferiority of TCA could not be shown for the PE at 4WFU, TCA was non-inferior to ECA at 24WFU (therapeutic success 50.8% vs. 48.7% (difference 2.2%, 95%CI -10.7% to 15.0%)). Adverse events occurred in 67.8% in both groups at 4WFU (mostly mild or moderate). Severe treatment-related pain was significantly less frequent in the TCA-group (4.2%) compared to the ECA-group (14.8%). HPV-findings did not differ between treatment groups between baseline and follow-up visits.

**Conclusion:**

TCA is an effective, well-tolerated and less complex alternative to ECA for the treatment of AIN in PLWH.

**Supplementary Information:**

The online version contains supplementary material available at https://doi.org/10.1007/s15010-026-02882-z.

## Introduction

Anal squamous cell carcinoma is induced by persistent infections with high-risk human papillomaviruses (HPV). Anal squamous cell carcinoma is preceded by precursor-lesions called high-grade squamous intraepithelial lesions (HSIL) or high-grade (HG) anal intraepithelial neoplasia (AIN) [1–3].

The incidence of anal squamous cell carcinoma in people living with HIV (PLWH), especially in men having sex with men (MSM), is more than forty-fold higher than in the general population [2, 4].

Despite the use of antiretroviral therapy (ART), anal squamous cell carcinoma is one of the most frequent non-AIDS-defining cancers in PLWH [4, 5]. Early diagnosis and treatment of HSIL/AIN significantly reduce the risk of anal squamous cell carcinoma in PLWH [6]. However, the rate of HSIL/AIN recurrence after treatment is high [7–12].

Currently, there is no gold standard for the treatment of AIN and the available options have rarely been evaluated in prospective randomised trials [7, 12–16]. The ablation of AIN with electrocautery (ECA) is the most frequently used therapeutic approach [6–12]. In a randomised controlled trial (RCT), ECA has been shown to be superior to topical treatment with imiquimod or fluorouracil for intra-anal lesions (low-grade intraepithelial lesions (LSIL) and HSIL), but AIN recurrence rates and side effects such as pain were substantial [12]. For ECA, local anaesthesia is often necessary prior to treatment. Local anaesthesia prolongs the duration of anoscopy, is painful for the patient, and alters the morphology of the anal canal, thereby making lesion localization more difficult. Moreover, ECA needs special equipment and performance by trained physicians. Accordingly, there is an unmet medical need for more effective and less complex treatment options for AIN with fewer side effects. Topical trichloroacetic acid (TCA) is an inexpensive, well-tolerated, physician-applied treatment for anogenital warts, that causes denaturation of HPV-DNA and cellular destruction by protein coagulation [17]. The use of TCA does not require any specific technical training. To date, TCA for AIN treatment has only been evaluated in three retrospective uncontrolled studies and one observational single-centre study that showed clearance rates comparable or superior to those reported for ECA, possibly with fewer side effects [8, 10, 18, 19].

The aim of this prospective RCT was to compare efficacy (clinical and histological response), safety, and impact on HPV-parameters of topical *T*CA-treatment versus ablation with *EC*A for the treatment of *AIN* in PLWH (TECAIN study).

## Patients and methods

### Study design and study participants

The TECAIN study was a prospective, open-label, multicentre, randomised (1:1), non-inferiority trial with two parallel treatment arms carried out in seven proctology units in Germany (Supplementary material).

Key inclusion criteria were an age of ≥ 18 years, written informed consent, confirmed HIV-infection, and histologically confirmed intraanal AIN (LSIL/HSIL) at study entry. Exclusion criteria were invasive anal cancer during the last five years, acute life-threatening disease, pregnancy, lactation, mental dysfunction, previous HPV vaccination, current topical or systemic treatment with immunomodulatory substances, serious proctologic disorders such as Crohn´s disease, or known intolerance against TCA or ECA.

The trial was conducted in accordance with the International Council for Harmonisation guidelines for Good Clinical Practice, the Declaration of Helsinki, and the professional code for physicians in Germany. It was approved by ethics committees and institutional review boards of all participating sites. All patients provided written informed consent before enrolment.

Prior to the start of the study, all centres that had applied to take part in the TECAIN study were assessed during a pre-study visit to determine their suitability for participation in the study. Only specialists trained in proctology, HRA, and treatment of AIN were eligible to serve as investigators to ensure a consistent approach according to the study protocol across all study centres. Following the enrolment of the first two patients and again as the study progressed, regular monitoring visits were carried out at all participating centres.

### Randomisation and masking

Eligible and consenting PLWH were randomly assigned to topical treatment with TCA or ablation with ECA. Digital central 1:1 block randomisation with variable block length was performed with the web-based randomisation service “TENALEA” [20] stratified by site. Masking of clinical investigators and patients was not possible because of obviously different treatment modalities. The pathologists assessing anal biopsies, the virologists performing HPV-analyses, the data safety monitoring board (DSMB), and the statisticians were blinded to the treatment modality. Unblinding occurred after data base lock. Clinical follow-up assessments at each site were performed by the same investigator who recruited the patients and informed them about their study allocation.

### Procedures

PLWH were screened for AIN (LSIL/HSIL) with high-resolution anoscopy (HRA)-guided intraanal biopsy collection and ECA and TCA as treatment options for AIN.

**Fig. 1:**
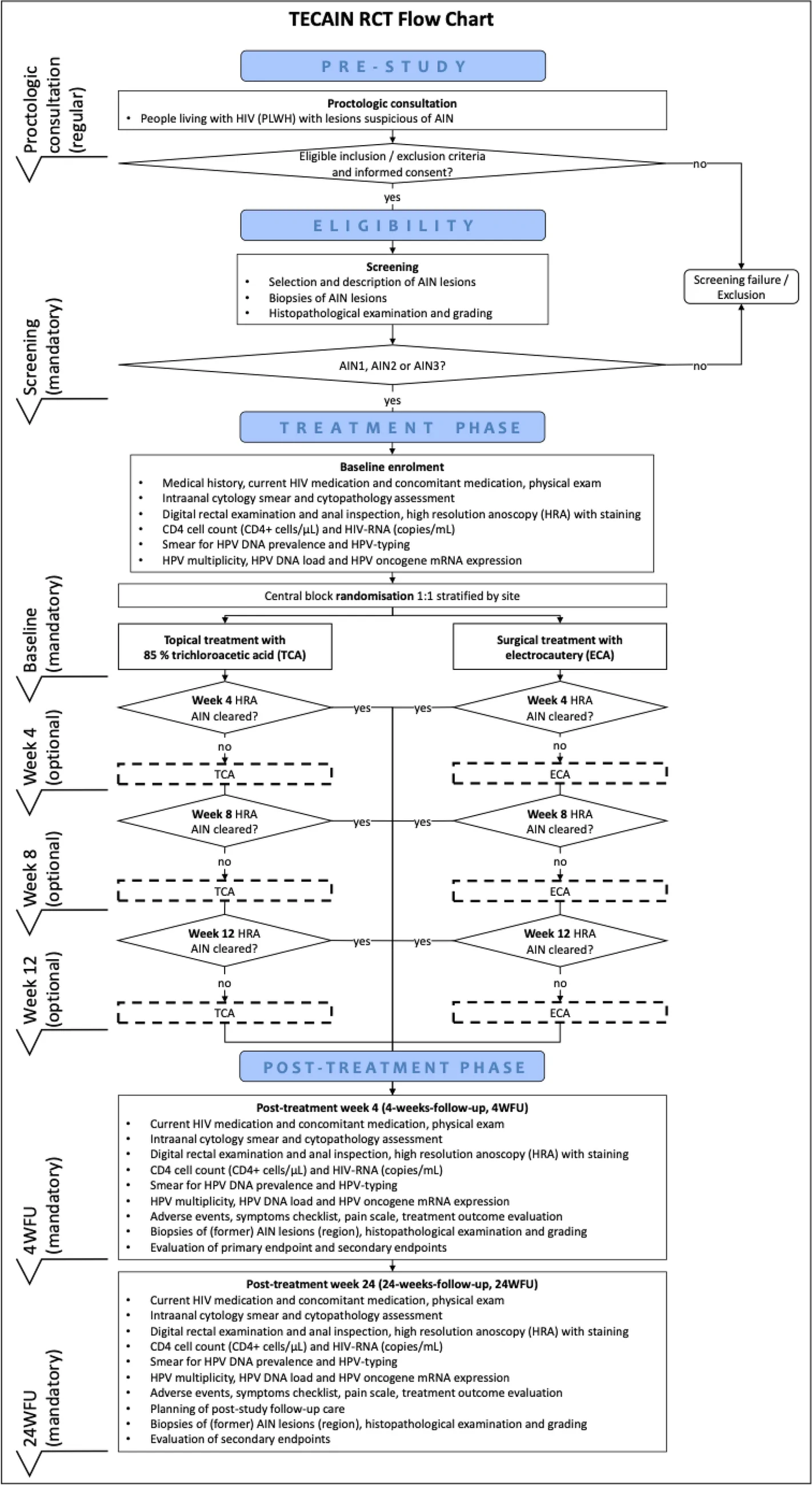
Graphical flow chart of the TECAIN randomised controlled trial. 4WFU, 4-weeks-follow-up; 24WFU, 24-weeks-follow-up; AIN, anal intraepithelial neoplasia; CD4, cluster of differentiation 4; DNA, deoxyribonucleic acid; ECA, electrocautery; HIV, human immunodeficiency virus; HPV, human papillomavirus; HRA, high-resolution anoscopy; mL, millilitre; µL, microlitre; mRNA, messenger ribonucleic acid; PLWH, people living with HIV; RCT, randomised controlled trial; RNA, ribonucleic acid; TCA, trichloroacetic acid

Diagnostic procedures at study visits included perianal inspection, swab collection for anal cytology and HPV-analyses, digital anorectal examination (DARE), and HRA with targeted biopsy collection of intraanal lesions suspicious of AIN (LSIL/HSIL) (Fig. 1). HRA was performed using 5% acetic acid and Lugol´s solution as described previously [21]. The number, location, and extent of AIN-suspicious lesions were documented on a case-report form (see Supplementary material). After histopathological confirmation, one to three AIN-lesions (LSIL/HSIL) were selected by the investigator as marker-lesion(s) (defined as the most severe lesion/s for which histopathological confirmation was available) to assess treatment outcome.

HRA-guided ECA- or TCA-treatment was conducted for all visible lesions at baseline. If residual AIN-lesions (LSIL/HSIL) were present, further optional identical treatment cycles were allowed every four weeks up to a maximum of three times after baseline treatment (weeks 4, 8, 12) (Fig. 1). All visible lesions were treated at each visit. If necessary, patients received local anaesthesia (lidocaine 1%), oral/intravenous sedatives, or general anaesthesia. Some patients could not tolerate the pain during HRA and AIN treatment and required sedation or general anaesthesia in addition to local anaesthesia. Patients for whom outpatient procedures under local anaesthesia were generally ruled out due to their sensitivity to pain were not supposed to be included in the study.

For ECA-treatment, ablation was performed using an electrosurgical unit with 2–5 mm spheres. For TCA-treatment, wooden-tipped cotton applicators were used to apply 85% (w/v) TCA-solution.

At each treatment visit and at the follow-up visits four and 24 weeks after the end of treatment, the presence and intensity (none/mild/moderate/severe) of self-reported and physician-reported adverse events (AE) were recorded.

### HPV analyses

Circumferential intraanal swabs for HPV analyses were collected in 3 mL ThinPrep-PreservCyt™ solution (Hologic, Marlborough/MA, USA) prior to DARE and HRA. For HPV-DNA and HPV-oncogene-mRNA analyses 1 mL was used, respectively. HPV-DNA detection, HPV-typing for 41 high-risk and low-risk HPV-types, and HPV-DNA load determination for ten high-risk-HPV-types (HPV16, 18, 31, 33, 45, 51, 52, 56, 58, 59) were performed as described previously [22]. HPV-DNA load was expressed as HPV-DNA copies per betaglobin gene copy. HPV-oncogene (E6/E7)-mRNA detection for 14 high-risk-HPV-types (HPV16, 18, 31, 33, 35, 39, 45, 51, 52, 56, 58, 59, 66, and 68) was performed with the Aptima™ HPV-assay on a DTS^TM^-system according to the manufacturer´s instructions (Hologic).

### Outcomes

The primary endpoint (PE) was therapeutic success defined as a combination of complete clinical response (CCR) evaluated by HRA and histologically confirmed clearance of AIN or regression/downgrade of HGAIN (HSIL, AIN2 + AIN3) to low-grade AIN (LSIL, AIN1) four weeks after the last treatment (4-weeks-follow-up, 4WFU). CCR was defined as clearance of any lesion at follow-up.

Key secondary endpoints (SE) were sustained therapeutic success (CCR + histological clearance of AIN or downgrade of HGAIN/HSIL to AIN1/LSIL) 24 weeks after the last treatment (24-weeks-follow-up, 24WFU), and CCR or histological clearance/downgrade at 4WFU and 24WFU, each evaluated separately. Recurrence rates of AIN were evaluated at 24WFU. Further SE were AE and the number of interventions during the 12-weeks treatment period. Anal HPV-DNA prevalence, HPV multiplicity (number of HPV-types), high-risk-HPV-DNA load, and HPV-oncogene-mRNA expression were compared between treatment groups at 4WFU and 24WFU, and within treatment groups at 4WFU and 24WFU in comparison to baseline, respectively.

### Statistical analysis

The primary hypothesis of the TECAIN study was that the efficacy of TCA is non-inferior to ECA. Based on previous studies, success rates of 50% were assumed for the PE in both treatment groups [12, 19]. On this basis and assuming that a 38% success rate is still acceptable, a non-inferiority margin of -12% was chosen. With a power of 80% and a significance level of α = 2.5%, the inclusion of 280 patients per group was calculated.

**Fig. 2:**
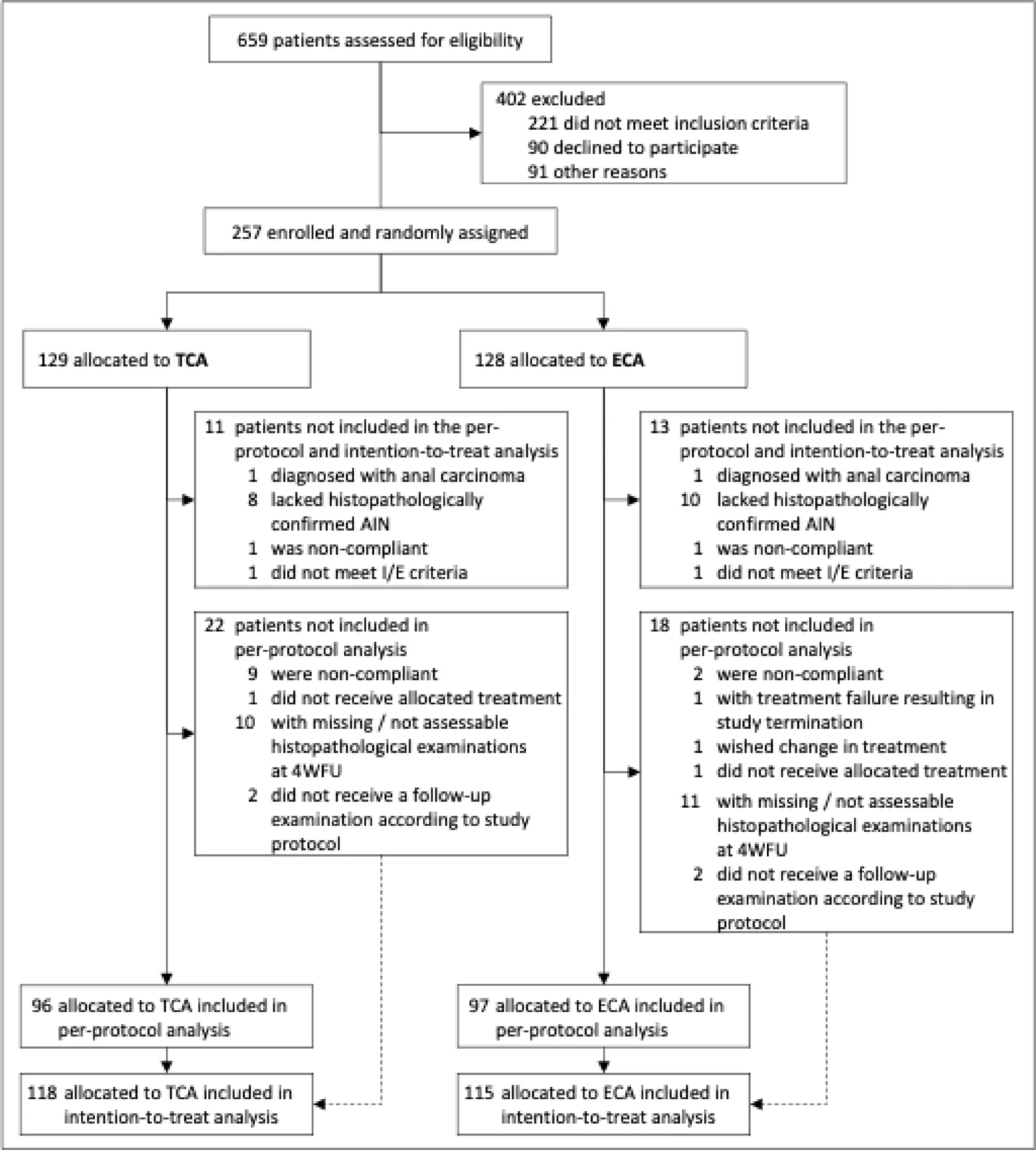
Trial profile of the TECAIN randomised controlled trial. AIN, anal intraepithelial neoplasia; ECA, electrocautery; I/E, inclusion/exclusion; TCA, trichloroacetic acid; 4WFU, 4-weeks-follow-up (4 weeks after the end of treatment)

Data were analysed in the intention-to-treat (ITT) population as primary analysis and in the per-protocol (PP) population as sensitivity analysis. The ITT analysis included all randomised patients who received at least one treatment with TCA or ECA. The PP analysis included all patients treated with ECA or TCA until 4WFU excluding patients with considerable protocol deviations (Fig. 2). Missing outcomes in the ITT-population were regarded as treatment failure.

In addition to the PE, non-inferiority tests were performed separately for histological clearance/downgrade and CCR. Non-inferiority was also tested at 24WFU in the ITT- and PP-population, and as a subgroup-analysis separately for patients with and without primary success at 4WFU. Further subgroup analyses distinguished between smokers and non-smokers, and patients with CD4-cell counts < 350 and > = 350 cells/µL at baseline.

To assess non-inferiority, two-sided 95% Wald confidence intervals (CI) were reported. The null hypothesis of inferiority was rejected with a significance level of α = 2.5%, if the lower confidence limit was greater than **−** 12%.

Baseline characteristics were described for the ITT-population using median, 25%- and 75%-quantiles for continuous variables, and number and percentage of occurrences for binary variables. AE were evaluated per patient and per intervention in the ITT-population at 4WFU. The DSMB met annually to review patient safety and data integrity.

Comparisons to baseline were analysed using two-sided McNemar’s test for binary outcomes and Wilcoxon signed-rank test for continuous outcomes. To compare treatment groups for binary outcomes, Fisher’s exact test was performed. Two-sided Mann-Whitney U test was applied to compare treatment groups for continuous outcomes. A p-value < 0.05 was considered statistically significant. Clopper-Pearson (exact binomial) 95%CIs were calculated for percentages and 95% Wald CIs were calculated for differences in percentage values (difference in proportion, Δ-proportion). All secondary analyses were conducted exploratory, so that no adjusting for multiple testing was performed. All statistical analyses were conducted using SAS software (version 9.4).

The trial is registered at ClinicalTrials.gov, NCT02615860 and the German Clinical Trials Register (DRKS), DRKS00009692.

## Results

### Patient recruitment and baseline characteristics

Between November 11, 2015, and March 31, 2020, 659 PLWH were assessed for eligibility. Histologically confirmed AIN of any grade (LSIL/HSIL) was diagnosed by HRA-targeted biopsies in 438 individuals. 257 of these patients met the inclusion criteria and were randomised (Fig. 2). 129 patients were allocated to TCA- and 128 to ECA-treatment. Due to protocol violations 11 patients of the TCA-group and 13 patients of the ECA-group did not receive the allocated intervention, leaving 233 patients in the ITT-population (118 TCA, 115 ECA). PP-analysis was done in 193 patients (96 TCA, 97 ECA) (see Fig. 2 for details).

Among the 233 participants in the ITT population, 229 (98.3%) were male, including 187 (80.3%) men who have sex with men (MSM). At baseline, participants´ median age was 48 years (IQR 40–54) and the median time since HIV-diagnosis was nine years (IQR 4–17). 60 (26.2%) PLWH had advanced HIV-infection (CDC-stage C) and 82 (35.8%) had a CD4 cell nadir below 200 cells/µL. Overall, 230 participants (98.7%) were receiving antiretroviral therapy (ART), with a mean treatment duration of 9.3 ± 7.3 years. Plasma HIV RNA was < 50 copies/mL in 207 participants (95.0%), and the median CD4 cell count was 653 cells/µL (IQR, 444–788). Eighty-five participants (36.5%) were current smokers. Prevalent sexually transmitted infections (STI) were diagnosed in 37 (15.9%) patients (Table 1). An evaluable anal cytology according to the Bethesda classification was observed in 211 participants, revealing HSIL in 74 (35.1%), LSI in 67 (31.8%), and atypical squamous cells of undetermined significance/atypical squamous cells, cannot exclude HSIL (ASC-US/ASC-H) in 48 (22.8%). Histopathologically confirmed AIN1/LSIL was found in 86 (36.9%) and HG-AIN (AIN2/AIN3, HSIL) in 147 (63.1%) participants. At baseline, HPV-DNA was found in 220 (96.5%) of 228 patients with evaluable anal swabs. High-risk-HPV-DNA was detected in 182 (79.8%,) HPV-oncogene-mRNA in 176 (77.2%) and HPV16 in 93 (40.8%) baseline swabs. The median number of HPV-types found per swab was two (IQR 2–4). With the exception of current STI, baseline characteristics were well balanced between the two treatment groups (Table 1).

**Table 1:** Baseline characteristics of the intention-to-treat (ITT) population

	Total (*n* = 233)	TCA (*n* = 118)	ECA (*n* = 115)
Sex (self-reported)			
Male	229 (98.3%)	117 (99.2%)	112 (97.4%)
Female	4 (1.7%)	1 (0.9%)	3 (2.6%)
Age (years)	48 (40–54)	48 (40–53)	49 (40–54)
Time since HIV diagnosis (years)	9 (4–17)	9 (4–16)	10 (4–18)
Mode of HIV infection (self-reported)			
MSM	187 (80.3%)	97 (82.2%)	90 (78.3%)
Heterosexual	12 (5.2%)	5 (4.2%)	7 (6.1%)
IVDU	4 (1.7%)	3 (2.5%)	1 (0.9%)
Unknown/other	30 (12.9%)	13 (11.0%)	17 (14.8%)
Current CD4 cell count, cells/µL	653 (444–788)	640 (440–778)	665 (463–816)
Missing	8	2	6
CD4 cell nadir category			
≥ 500 CD4-cells/µL	51 (22.3%)	32 (27.8%)	19 (16.7%)
≥ 200 < 500 CD4-cells/µL	96 (41.9%)	44 (38.3%)	52 (45.6%)
< 200 CD4-cells/µL	82 (35.8%)	39 (33.9%)	43 (37.7%)
Missing	4	3	1
HIV-1 RNA-load in plasma, copies/mL	10 (0–20)	10 (0–20)	10 (0–20)
Undetectable HIV-1 RNA, < 50 copies/mL	207 (95.0%)	106 (93.0%)	101 (97.1%)
Detectable HIV-1 RNA, ≥ 50 copies/mL	11 (5.0%)	8 (7.0%)	3 (2.9%)
Missing	15	4	11
CDC category of HIV infection^a^			
A	98 (42.8%)	51 (44.3%)	47 (41.2%)
B	71 (31.0%)	35 (30.4%)	36 (31.6%)
C	60 (26.2%)	29 (25.2%)	31 (27.2%)
Missing	4	3	1
Currently using ART	230 (98.7%)	116 (98.3%)	114 (99.1%)
Smoking			
Non-smoker	148 (63.5%)	70 (59.3%)	78 (67.8%)
Current smoker	85 (36.5%)	48 (40.7%)	37 (32.2%)
Any current STI^b^	37 (15.9%)	12 (10.2%)	25 (21.7%)
Anal cytology			
NILM	22 (10.4%)	15 (14.2%)	7 (6.7%)
ASC-US/ASC-H	48 (22.8%)	23 (21.7%)	25 (23.8%)
LSIL	67 (31.8%)	31 (29.3%)	36 (34.3%)
HSIL	74 (35.1%)	37 (34.9%)	37 (35.2%)
SCC	0	0	0
Missing/not evaluable	22	12	10
Histological AIN grade			
AIN1	86 (36.9%)	40 (33.9%)	46 (40.0%)
AIN2	70 (30.0%)	38 (32.2%)	32 (27.8%)
AIN3	77 (33.1%)	40 (33.9%)	37 (32.2%)
HPV detection in anal swabs			
Any HPV-type^c^	220 (96.5%)	111 (95.7%)	109 (97.3%)
High-risk HPV-types^c^	182 (79.8%)	93 (80.2%)	89 (79.5%)
HPV16	93 (40.8%)	44 (37.9%)	49 (43.8%)
No. of HPV-types^d^, median (IQR), maximum	2 (2–4), 12	3 (2–3), 12	2 (1.5-4), 12
HPV-DNA negative	8 (3.5%)	5 (4.3%)	3 (2.7%)
HPV-oncogene-mRNA detection^e^	176 (77.2%)	90 (77.6%)	86 (76.8%)
HR-HPV-DNA load, median (IQR)^f^	19.2 (0.9-123.6)	15.5 (0.9-178.8)	20.4 (0.9–99.6)
Missing/not evaluable^g^	5	2	3

Data are n (%) or median (IQR). Percentages may not total 100 due to rounding

ART, antiretroviral therapy; ASC-H, atypical squamous cells, cannot exclude HSIL; ASCUS, atypical squamous cell of undetermined significance; ECA, electrocautery; HPV, human papillomavirus; HSIL, high grade squamous intraepithelial lesion; IVDU, intravenous drug use; LSIL, low grade squamous intraepithelial lesion; MSM, men having sex with men; NILM, negative for intraepithelial lesion or malignancy (normal cytology) ; SCC, squamous cell carcinoma; STI, sexually transmitted infection; TCA, trichloroacetic acid

^a^ The CDC category of HIV infection is given according to the 1993 revised classification system for HIV infection and expanded surveillance case definition for AIDS among adolescents and adults: https://www.cdc.gov/mmwr/preview/mmwrhtml/00018871.htm

^b^ Current STI: all patients were tested for Neisseria gonorrhoea and Chlamydia trachomatis by PCR of anal swabs and for Treponema pallidum infection (syphilis) by serology at baseline. Test for other STI (e.g. HSV-1/2, Haemophilus ducrei) were only performed in cases of clinical suspicion

^c^ Any HPV-type includes 41 high-risk, possible high-risk and low-risk HPV-types: HPV6, 11, 16, 18, 26, 30, 31, 33, 34, 35, 39, 40, 42, 43, 44, 45, 51, 52, 53, 54, 55, 56, 57, 58, 59, 61, 66, 67, 68, 69, 70, 71, 72, 73, 81, 82, 83, 84, 85, 89, 97 (for more details see Table S2, Supplementary material). High-risk HPV-types include HPV16, 18, 31, 33, 35, 39, 45, 51, 52, 56, 58, 59, and 68

^d^ No. of HPV-types includes all 41 HPV-types analysed in the study

^e^ HPV-oncogene-mRNA detection comprises the qualitative detection of viral oncogene (E6/E7) messenger RNA (mRNA) from 13 high-risk-HPV-types (16, 18, 31, 33, 35, 39, 45, 51, 52, 56, 58, 59, 68) and 1 probable high-risk-HPV-type (HPV66). The assay (Aptima HPV assay, Hologic) does not discriminate between the 14 HPV-types included

^f^ HPV16 + 18+31 + 33 + 45 + 51+52 + 56+58 + 59 cumulative DNA load (for details see Table S2, Supplementary material)

^g^ Samples in which no cellular DNA was detectable (betaglobin-gene PCR negative) were considered not evaluable with regard to HPV status

### Outcome of treatment

**Fig. 3:**
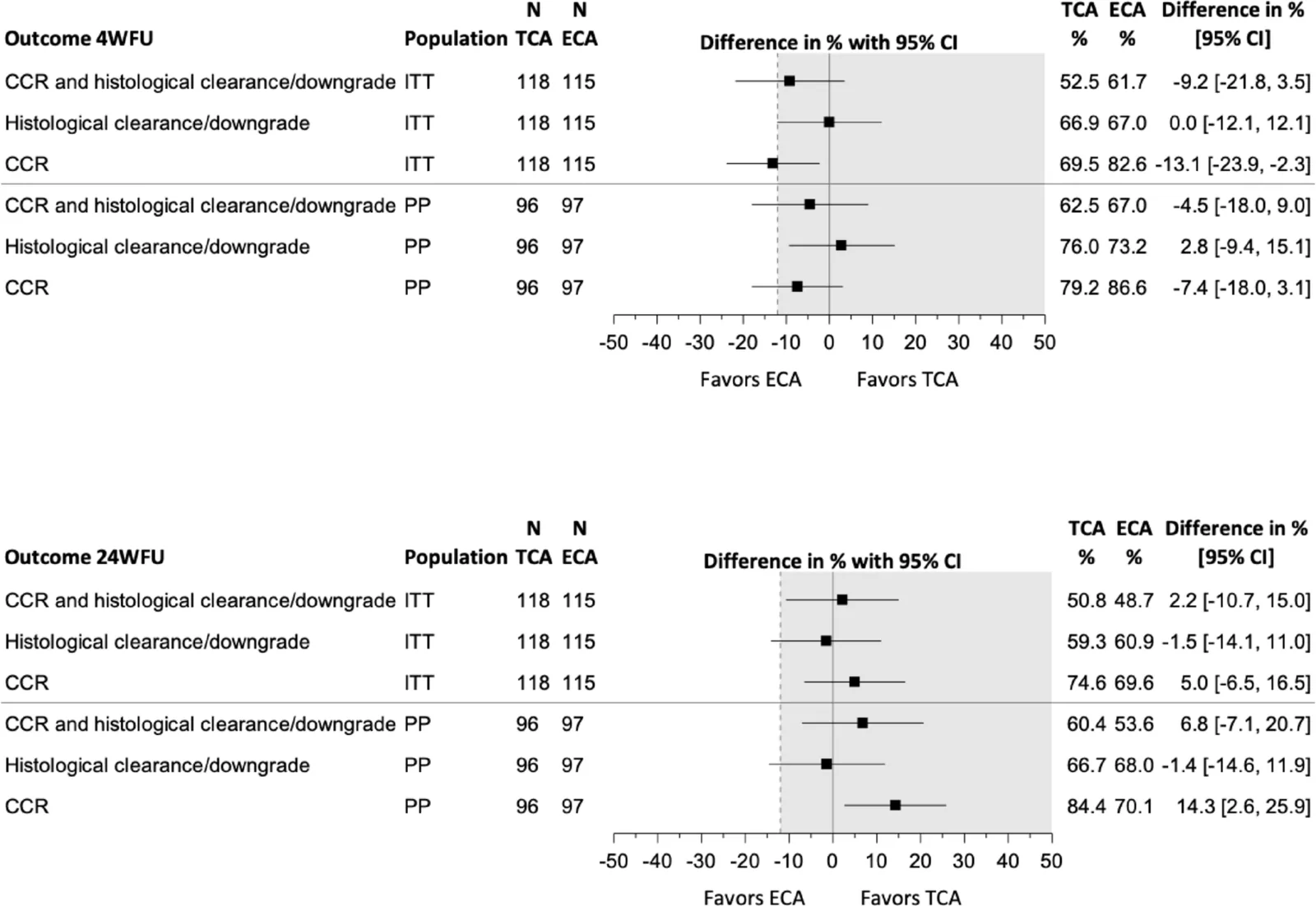
Forest plots showing the primary endpoint, histological clearance/downgrade, and complete clinical response (CCR) at 4-weeks-follow-up (4WFU, top, Fig. 3a) and at 24-weeks-follow-up (24WFU, bottom, Fig. 3b), ITT and PP

The PE of combined CCR and histologically confirmed clearance or downgrade of AIN marker-lesions at 4WFU was achieved in the ITT-analysis in 62 of 118 (52.5%) participants of the TCA-group and in 71 of 115 (61.7%) of the ECA-group (Fig. 3a). With the non-inferiority threshold of -12%, non-inferiority of TCA to ECA could not be shown at 4WFU (Δ-proportion − 9.2%, 95%CI -21.8% to 3.5%). Similar results were found for the PE in the PP-analysis (62.5% (TCA) vs. 67.0% (ECA); Δ-proportion − 4.5%, 95%CI -18.0% to 9.0%). Histological clearance/downgrade of the marker-lesions at 4WFU was almost identical in TCA- and ECA-treated patients in the ITT-analysis (TCA 66.9% vs. ECA 67.0%; Δ-proportion 0.0%, 95%CI -12.1% to 12.1%). Non-inferiority of TCA vs. ECA concerning histological clearance/downgrade at 4WFU could be shown in the PP-analysis (TCA 76.0% vs. ECA 73.2%; Δ-proportion 2.8%, 95%CI -9.4% to 15.1%). Concerning only CCR at 4WFU, non-inferiority of TCA vs. ECA was neither shown in the ITT- (TCA 69.5% vs. ECA 82.6%; Δ-proportion − 13.1%, 95%CI -23.9% to -2.3%) nor in the PP-analysis (TCA 79.2% vs. ECA 86.6%; Δ-proportion − 7.4%, 95%CI -18.0% to 3.1%) (Fig. 3a).

During the treatment-period of maximally 12 weeks (Fig. 1), study participants were treated on average 2.5 times (± 1.2 standard deviation (SD)). More interventions were required in the TCA-group than in the ECA-group for CCR (TCA: 2.8 (± 1.2 SD) vs. ECA: 2.3 (± 1.2 SD) interventions; *p* = 0.005).

In contrast to 4WFU, non-inferiority of TCA vs. ECA was shown at 24WFU with the non-inferiority threshold of -12% both in the ITT- and the PP-analysis for the combined endpoint of CCR and histological clearance/downgrade (ITT: TCA 50.8% vs. ECA 48.7%; Δ-proportion 2.2%, 95%CI -10.7% to 15.0%; PP: TCA 60.4% vs. ECA 53.6%; Δ-proportion 6.8%, 95%CI -7.1% to 20.7%) and for CCR alone (Fig. 3b).

Forest plots of the absolute differences in % of the two treatment groups TCA and ECA at 4WFU (top panel) and at 24WFU (bottom panel) each for the ITT and PP population with the following three outcomes (from top to bottom, respectively): (1) complete clinical response (CCR) and histological clearance/downgrade, (2) histological clearance/downgrade, and (3) CCR. The primary endpoint of the study is the combination of CCR (evaluated by HRA) and histological clearance/downgrade at 4WFU. The vertical dashed black line and the grey area right of the line represent the non-inferiority margin and the area above the margin of -12%. The displayed differences in % are based on the exact differences rounded after subtraction. Because of rounding, the displayed differences can differ slightly (+/- 0.1%) from the differences of the shown TCA minus ECA response rates.

CI, confidence interval; CCR, complete clinical response; ECA, electrocautery; ITT, intention-to-treat; N, number of patients; PP, per-protocol; TCA, trichloroacetic acid; 4WFU, 4-weeks-follow-up (4 weeks after the end of treatment); 24WFU, 24-weeks-follow-up (24 weeks after the end of treatment).

**Fig. 4:**
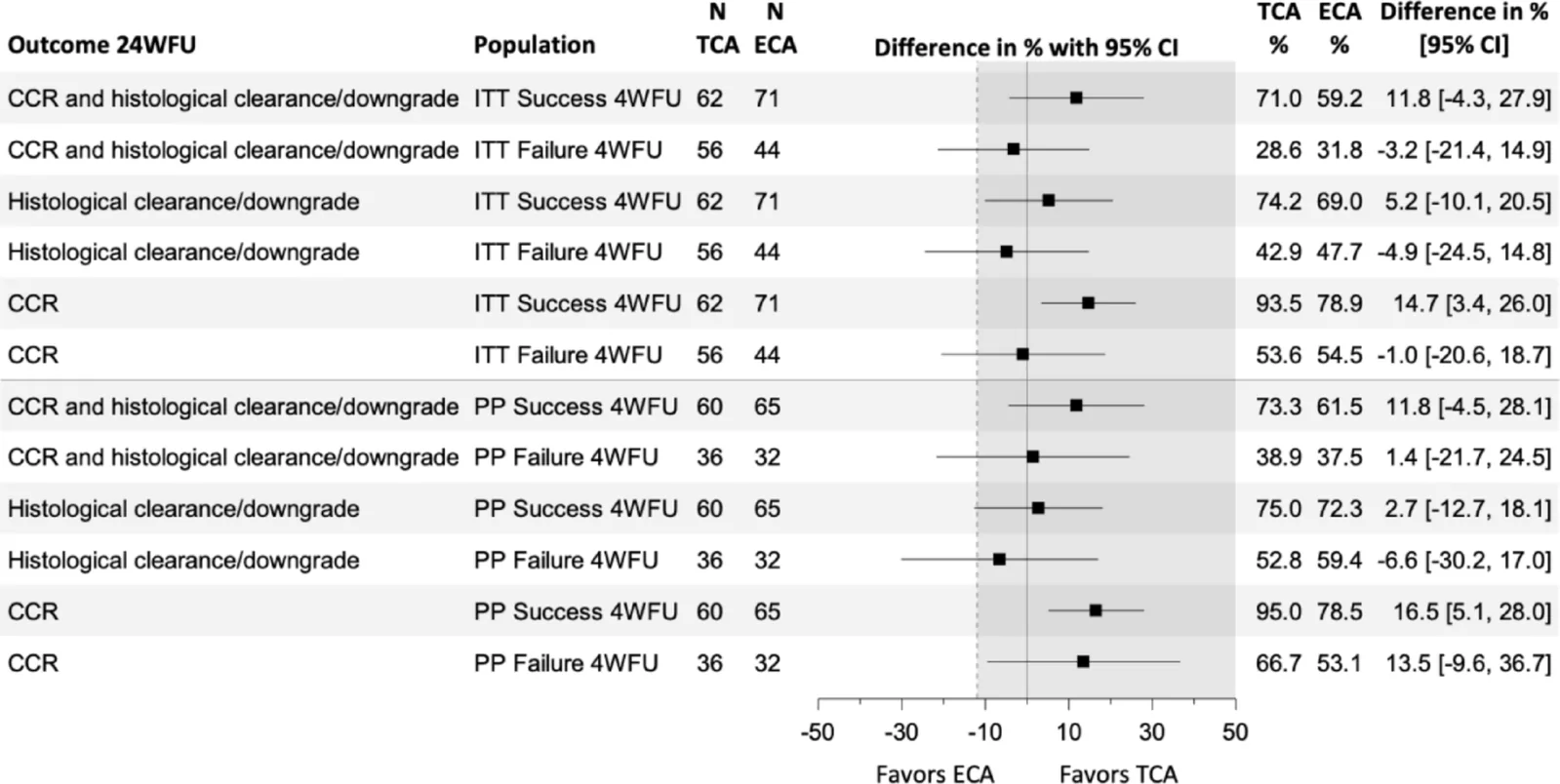
Outcome at 24-weeks-follow-up (24WFU) in patients with treatment success or treatment failure at 4-weeks-follow-up (4WFU), ITT and PP

Figure 4 shows the outcome at 24WFU divided into patients who had reached or not reached the PE at 4WFU. Of the 133 participants in the ITT-population who achieved the PE at 4WFU (62 in the TCA-and 71 in the ECA-group), 86 (64.7%) subsequently achieved the combined endpoint of CCR and histological clearance/downgrade at 24WFU. The proportion was higher in the TCA group than in the ECA group (71.0% vs. 59.2%; Δ-proportion 11.8%, 95%CI -4.3% to 27.9%). After evaluation at 4WFU the study protocol allowed further treatments chosen by the investigator (without any restrictions on treatment options) in participants who had not reached the PE. Of these 100 individuals (TCA-group *n* = 56, ECA-group *n* = 44), 28.6% of the TCA-group and 31.8% of the ECA-group (Δ-proportion − 3.2%, 95%CI -21.4% to 14.9%) reached CCR and histological clearance/downgrade at 24WFU. Non-inferiority of TCA vs. ECA at 24WFU could be shown for all outcomes for patients with primary success at 4WFU in the ITT-population. Accordingly, patients with treatment success (histology and/or CCR) at 4WFU had much lower recurrence rates in both treatment groups at 24WFU than those with treatment failure at 4WFU. In patients with treatment success at 4WFU, recurrence rates at 24WFU were lower in the TCA- than in the ECA-group: the AIN recurrence rate (CCR + histological) at 24WFU in the 133 ITT-patients with treatment success at 4WFU was 35.3% in total, 29.0% in the TCA-group compared to 40.8% in the ECA-group (Δ-proportion − 11.8%, 95%CI -27.9% to 4.3%). The respective numbers in the PP-population were 26.7% (TCA) and 38.5% (ECA) (Δ-proportion − 11.8%, 95%CI -28.1% to 4.5%). None of the 233 ITT-patients developed invasive intraanal squamous cell carcinoma until 24WFU.

Forest plots of the absolute differences in % of the two treatment groups TCA and ECA at 24WFU, differentiating between patients who had treatment success (dark grey background) or treatment failure (light grey background) at 4WFU, for the ITT (top panel) and PP (bottom panel) population.

The following three outcomes are shown from top to bottom, respectively: (1) complete clinical response (CCR) and histological clearance/downgrade, (2) histological clearance/downgrade, and (3) CCR. The vertical dashed black line and the grey area right of the line represent the non-inferiority margin and the area above the margin of -12%. The displayed differences in % are based on the exact differences rounded after subtraction. Because of rounding, the displayed differences can differ slightly (+/- 0.1%) from the differences of the shown TCA minus ECA response rates.

CI, confidence interval; CCR, complete clinical response; ECA, electrocautery; ITT, intention-to-treat; N, number of patients; PP, per-protocol; TCA, trichloroacetic acid; 4WFU, 4-weeks-follow-up (4 weeks after the end of treatment); 24WFU, 24-weeks-follow-up (24 weeks after the end of treatment).

### The effect of smoking and CD4-cell count at baseline on outcome of treatment

No association between treatment response and smoking could be detected at 4WFU and 24WFU (Fig. S1, Supplementary material). Regarding CD4-cell counts at baseline, outcomes between patients with < 350 vs. >=350 CD4-cells/µL were similar at 4WFU. At 24WFU, non-inferiority of TCA vs. ECA could only be shown in patients with > = 350 CD4-cells/µL at baseline (Fig. S2, Supplementary material).

### Adverse events

AE were balanced in the two treatment groups. Until 4WFU, one or more AE were documented in both treatment arms for 67.8% of the patients (ITT-population: TCA 80/118, ECA 78/115). Most AE were considered treatment-related and were mild and temporary (Table 2). The most frequent patient-reported AE were pain followed by burning. Severe pain occurred less frequently in the TCA- than in the ECA-group (4.2% vs. 14.8%; Δ-proportion − 10.5%, 95%CI -18.0% to -3.1%) and mild burning was reported more frequently in the TCA-group. When AE were evaluated per intervention, pain of any grade was less frequent in the TCA- compared to the ECA-group (21.0% (68/324) vs. 36.2% (96/265); Δ-proportion − 15.2%, 95%CI -22.5% to -7.9%) (Table S1, Supplementary material). Anaesthesia was needed less frequently in the TCA-group compared to the ECA-group, both when analysed per patient (8.5% vs. 34.8%; Δ-proportion − 26.3%, 95%CI -36.4% to -16.3%) and per intervention (8.6% vs. 39.6%; Δ-proportion − 31.0%, 95%CI -37.6% to -24.3%). With the exception of bleeding during or directly after treatment, physician-reported AE such as wound healing disorder, fissure formation, or wound infection did not occur in the TCA-group, but were documented in two patients of the ECA-group. Scar formation, anal stenosis, or allergic reactions did not occur in any patient. Four serious adverse events (SAE) occurred in each treatment group. None of them was considered treatment-related (Table 2). Between 4WFU and 24WFU, no further AE classified as study-treatment-related were reported.

**Table 2:** Adverse events (AE) of patients in the period from baseline to four weeks after the end of treatment (4WFU) in the intention-to-treat (ITT) population

	TCA (*n* = 118)	ECA (*n* = 115)	Difference of AE percentage values (95% CI)
Any AE, highest grade	80 (67.8%)	78 (67.8%)	0.0 (– 12.0 to 12.0)
None	38 (32.2%)	37 (32.2%)	0.0 (– 12.0 to 12.0)
Grade 1 (mild)	39 (33.1%)	26 (22.6%)	10.4 (– 1.0 to 21.9)
Grade 2 (moderate)	30 (25.4%)	32 (27.8%)	– 2.4 (– 13.8 to 8.9)
Grade 3 (severe)	11 (9.3%)	20 (17.4%)	– 8.1 (– 16.8 to 0.6)
Treatment-related AE,^a^ highest grade	76 (64.4%)	75 (65.2%)	– 0.8 (– 13.1 to 11.5)
Pain	47 (39.8%)	58 (50.4%)	– 10.6 (– 23.3 to 2.1)
None	71 (60.2%)	57 (49.6%)	10.6 (– 2.1 to 23.3)
Grade 1 (mild)	28 (23.7%)	24 (20.9%)	2.9 (– 7.8 to 13.5)
Grade 2 (moderate)	14 (11.9%)	17 (14.8%)	– 2.9 (– 11.6 to 5.8)
Grade 3 (severe)	5 (4.2%)	17 (14.8%)	– 10.5 (– 18.0 to – 3.1)
Burning	35 (29.7%)	28 (24.3%)	5.3 (– 6.1 to 16.7)
None	83 (70.3%)	87 (75.7%)	– 5.3 (– 16.7 to 6.1)
Grade 1 (mild)	23 (19.5%)	11 (9.6%)	9.9 (1.0 to 18.9)
Grade 2 (moderate)	10 (8.5%)	12 (10.4%)	– 2.0 (– 9.5 to 5.6)
Grade 3 (severe)	2 (1.7%)	5 (4.3%)	– 2.7 (– 7.0 to 1.7)
Itching	21 (17.8%)	11 (9.6%)	8.2 (– 0.5 to 17.0)
None	97 (82.2%)	104 (90.4%)	– 8.2 (– 17.0 to 0.5)
Grade 1 (mild)	17 (14.4%)	9 (7.8%)	6.6 (– 1.4 to 14.6)
Grade 2 (moderate)	4 (3.4%)	1 (0.9%)	2.5 (– 1.2 to 6.2)
Grade 3 (severe)	0 (0.0%)	1 (0.9%)	– 0.9 (– 2.6 to 0.8)
Spasms	7 (5.9%)	4 (3.5%)	2.5 (– 3.0 to 7.9)
None	111 (94.1%)	111 (96.5%)	– 2.5 (– 7.9 to 3.0)
Grade 1 (mild)	4 (3.4%)	1 (0.9%)	2.5 (– 1.2 to 6.2)
Grade 2 (moderate)	2 (1.7%)	2 (1.7%)	0.0 (– 3.4 to 3.3)
Grade 3 (severe)	1 (0.8%)	1 (0.9%)	0.0 (– 2.4 to 2.3)
Irregular bowel movements^b^	34 (28.8%)	28 (24.3%)	4.5 (– 6.9 to 15.8)
None	84 (71.2%)	87 (75.7%)	– 4.5 (– 15.8 to 6.9)
Grade 1 (mild)	27 (22.9%)	24 (20.9%)	2.0 (– 8.6 to 12.6)
Grade 2 (moderate)	4 (3.4%)	4 (3.5%)	– 0.1 (– 4.8 to 4.6)
Grade 3 (severe)	3 (2.5%)	0 (0.0%)	2.5 (– 0.3 to 5.4)
Bleeding^c^	5 (4.2%)	8 (7.0%)	– 2.7 (– 8.6 to 3.2)
None	113 (95.8%)	107 (93.0%)	2.7 (– 3.2 to 8.6)
Grade 1 (mild)	3 (2.5%)	3 (2.6%)	– 0.1 (– 4.1 to 4.0)
Grade 2 (moderate)	2 (1.7%)	5 (4.3%)	– 2.7 (– 7.0 to 1.7)
Grade 3 (severe)	0 (0.0%)	0 (0.0%)	0.0
Other treatment-related AE^d^	0 (0.0%)	2 (1.7%)	– 1.7 (– 4.1 to 0.7)
Median no. of treatment-related AE per patient (IQR)	1 (0–4)	2 (0–3)	*p* = 0.07^e^
Non-treatment-related AE	22 (18.6%)	18 (15.7%)	3.0 (– 6.7 to 12.7)
SAE^f^	4 (3.4%)	4 (3.5%)	– 0.1 (– 4.8 to 4.6)
Treatment-related SAE	0 (0.0%)	0 (0.0%)	0.0
Study discontinuation due to SAE^f^	0 (0.0%)	1 (0.9%)	– 0.9 (– 2.6 to 0.8)
Anaesthesia use	10 (8.5%)	40 (34.8%)	– 26.3 (– 36.4 to – 16.3)
None	108 (91.5%)	75 (65.2%)	26.3 (16.3 to 36.4)
Local	5 (4.2%)	32 (27.8%)	– 23.6 (– 32.5 to – 14.6)
Systemic sedatives	5 (4.2%)	7 (6.1%)	– 1.8 (– 7.5 to 3.8)
General anaesthesia^g^	0 (0.0%)	1 (0.9%)	– 0.9 (– 2.6 to 0.8)

AE, adverse event; ECA, electrocautery; IQR, interquartile range; SAE, serious adverse event; TCA, trichloroacetic acid

Unless otherwise stated data are in n (%). Percentage figures for AE that were significantly different between the TCA- and the ECA-group are printed in bold. Percentages may not total 100 due to rounding

4WFU data was available for 221 patients (109 for TCA, 112 for ECA). Between baseline and 4WFU nine patients in the TCA- and three patients in the ECA-arm dropped out of the study. Reasons were noncompliance (*n* = 9, all TCA), insufficient treatment efficacy (*n* = 1, ECA) and failure to attend 4WFU (*n* = 2, ECA)

^a^ Treatment-related self-reported AE include pain, burning, itching, spasms, irregular bowel movements, and bleeding. Physician-reported AE include bleeding, wound healing disorder, wound infection, scarring, fissure formation, anal stenosis, or allergic reactions. In terms of severity, the highest grade reported up to 4WFU was taken into account

^b^ Irregular bowel movements comprise obstipation, rectal discharge, faecal deposits, thin faeces, urge to defecate, diarrhoea and flatulence

^c^ Bleeding comprises both bleeding reported by the patient and bleeding during or directly after treatment reported by the physician

^d^ Other treatment-related AE in two patients of the ECA-group were post-operative wound healing disorder (grade 2) and fissure formation after the last treatment (grade 3) in one patient and a post-operative wound infection after the last treatment in another patient (grade 2). Scar formation, anal stenosis, or allergic reactions did not occur in any patient

^e^ Wilcoxon test

^f^ The four SAE in the TCA-group were recurrent herniated intervertebral discs, ear surgery, benign prostate hyperplasia, and hospitalisation for surgical removal of anogenital warts four weeks after AIN-treatment. The four SAE in the ECA-group were angina pectoris (four weeks after the end of treatment), an apoplectic stroke (three weeks after the end of treatment), multi-substance abuse, and hospitalisation for surgical removal of expanding intraanal AIN. The patient with the latter SAE discontinued the study due to hospitalisation for surgical removal of the expanding AIN three weeks after the baseline visit. Each of the eight SAEs mentioned from both groups occurred in only one patient. All SAE were not considered treatment-related

^g^ General anaesthesia was only necessary for one ECA intervention (and was never applied in the TCA group). Systemic sedatives included oral or intravenous medication for sedation

See Table S1 in the supplementary material for AEs in relation to the number of interventions.

### Impact of treatments on HPV findings

HPV parameters did not differ between the treatment groups at baseline, 4WFU or 24WFU, respectively. Neither TCA- nor ECA-treatment led to significant changes of HPV-DNA prevalence, HPV-oncogene-mRNA detection or HPV-DNA-load (Tables S2 + S3, Supplementary material). Only the number of high-risk- or low-risk-HPV-types decreased between baseline and 4WFU or 24WFU in both treatment arms of the ITT-population (*p* = 0.01 or *p* = 0.02; Table S2, Supplementary material).

## Discussion

Current guidelines recommend anal cancer screening and treatment of precursor-lesions in PLWH [2, 23, 24]. Because of high recurrence rates, treatment of AIN is challenging in PLWH [6]. ECA is the most commonly used method for AIN-treatment [6, 7]. However, ECA has disadvantages, including the need for technical equipment and frequent intraoperative pain [6, 7, 12, 16]. Compared to ECA, topical approaches as imiquimod, 5-fluorouracil, cidofovir, and sinecatechins needed more applications, showed reduced effectiveness, had higher recurrence rates, and were more frequently associated with side effects [7, 12]. In retrospective uncontrolled and observational studies TCA for the treatment of AIN (LSIL/HSIL) has shown promising results [8, 10, 18, 19]. To our knowledge, the TECAIN-study is the first RCT investigating TCA vs. ECA for the treatment of AIN (LSIL/HSIL) [16].

Although the PE of non-inferiority of TCA vs. ECA was not reached at 4WFU, the TECAIN-study demonstrated that TCA was non-inferior to ECA concerning CCR and histological clearance/downgrade 24 weeks after the end of treatment.

The efficacy of TCA for the treatment of AIN (LSIL/HSIL) has been demonstrated in four uncontrolled studies [8, 10, 18, 19]. The response rates of TCA in these trials were higher (around 70–80%) than in our study. The different response rates can be partly explained by differences in study design, patient characteristics, and outcome measures.

In previous studies ECA and TCA were well tolerated [6, 8–10, 12, 19]. Also in our study, no permanent AE and no treatment-related SAE were observed in either treatment group. Although anaesthetics were used more frequently in the ECA- than in the TCA-group, treatment-related severe pain occurred significantly more often in the ECA-group. The better tolerability of TCA could make the repeated applications often necessary for AIN (LSIL/HSIL) resolution more acceptable to patients than with ECA.

Progression to invasive cancer is a concern in treatment studies on anogenital HSIL [6, 25]. In our study, progression to anal squamous cell carcinoma was not observed at treated sites, but the follow-up period was only 24 weeks.

Apart from the number of high-risk and low-risk-HPV-types, both treatment modalities had no impact on HPV-parameters. This is different from the findings by Burgos and colleagues who reported a decrease of high-risk-HPV/HPV16-prevalence after TCA-treatment (but not after ECA-treatment) [8]. Different statistical approaches or random differences could explain the different results. Furthermore, TCA is an ablative treatment that is not expected to change HPV-parameters in the anal canal. Our findings argue against comprehensive HPV-testing in the context of AIN-treatment in PLWH.

Strengths of our study include the prospective design, randomisation, high adherence of randomised patients, rigorous documentation of AIN marker-lesions, HRA-guided and histopathology-based inclusion criteria and endpoints, and comprehensive HPV-analyses. To the best of our knowledge, the TECAIN-study is the first RCT comparing TCA with ECA for the treatment of AIN (LSIL/HSIL) [16]. There is a medical need for topical agents such as TCA that demonstrate comparable long-term outcomes to ECA in RCTs and are both inexpensive and globally available. Nevertheless, the TECAIN-study has limitations: (1) Due to slow patient recruitment, the study period was longer and the number of patients randomised was lower than planned. The latter may have led to lower statistical power to demonstrate non-inferiority of TCA with a threshold of -12% at 4WFU. With 233 patients with an evaluable PE, the TECAIN-study is the largest RCT comparing different treatment interventions for AIN (LSIL/HSIL) in PLWH. The estimated success rate for the PE in the TCA-group was 52.5% in the ITT-population and 62.5% in the PP-population, which was well above the predefined threshold for therapeutic success of 38%. Non-inferiority of TCA was demonstrated at 24WFU using the criteria defined for the PE. (2) Only PLWH (98% males) without HPV-vaccination were included. Therefore, our results cannot be generalised to patients without HIV-infection, females, transgender persons, and HPV-vaccinated individuals. (3) Measurement bias regarding treatment-related AE is possible, because more time points for interventions were required in the TCA- than in the ECA-group. (4) Neither the assessors of clinical outcomes nor the patients were blinded to the applied study treatment. 5) If anal HPV-lesions present at baseline were missed during the initial HRA (underdiagnosis), their subsequent detection at the 24-week follow-up could be erroneously classified as recurrence or new disease rather than persistent disease. 6.) Finally, the whole spectrum of AIN (LSIL/HSIL) was included. This is both a strength and a limitation of the study. Most current recommendations suggest only treatment of HGAIN/HSIL [23], but some guidelines and previous studies also included low-grade AIN/LSIL [12, 19, 26]. Although low-grade AIN/LSIL can regress without treatment [27], in many countries (e.g. Germany) all clinically AIN-suspected lesions are treated immediately before histological results are available, due to scarce HRA capacities.

AIN (LSIL/HSIL) and anal squamous cell carcinoma can be prevented by prophylactic HPV vaccination [28, 29]. Until the full impact of HPV vaccination can be seen in all age-groups, screening and treatment of AIN (LSIL/HSIL) remain the main pillars of anal cancer prevention in high-risk groups [2, 6, 23, 24].

## Conclusion

TCA was non-inferior to ECA for the treatment of AIN (LSIL/HSIL) in PLWH at 24WFU. Advantages of TCA comprise practicability in recurrent AIN, little treatment-associated pain, no regular need of local anaesthesia, less technical equipment, no risk to health care workers from surgical smoke, and low costs [8, 17, 19]. Therefore, TCA could facilitate the implementation of anal squamous cell carcinoma screening and treatment of AIN (LSIL/HSIL) in PLWH, even in low-resource settings. Anal squamous cell carcinoma incidence is increasing in Europe and North America [5, 30]. Apart from PLWH, iatrogenically immunosuppressed patients and women with genital (pre)cancers are high-risk groups for anal squamous cell carcinoma [4]. Therefore, future research should investigate TCA for the treatment of AIN in these groups. TCA is an easy-to-use, well-tolerated and inexpensive alternative to electrocautery in PLWH.

## Supplementary Information

Below is the link to the electronic supplementary material.

Supplementary Material 1

## Data Availability

Data availabilityThe data that support the findings of this study are available from the corresponding author upon reasonable request.
