# Supplementary material for: Topical trichloroacetic acid versus electrocautery for treatment of anal intraepithelial neoplasia in people living with HIV: a multicentre, randomised, non-inferiority trial (TECAIN-study)
Source: Infection. 2026 Jul 10;54(5):2563–77. doi: 10.1007/s15010-026-02882-z (PMC13727835; doi:10.1007/s15010-026-02882-z)
Supplement: Supplementary file 1 [file 15010_2026_2882_MOESM1_ESM.pdf]

Supplementary Information

Supplement to Stefan Esser*, Alexander Kreuter, Anja Potthoff, Robert Jablonka, Mark Oette, Martin F. Sprinzl, Daniel Exner, Konstantinos Bilbilis, Hildegard Lax, Eva-Maria Huessler, Steffi Silling, Ulrike Wieland: Topical trichloroacetic acid versus electrocautery for treatment of anal intraepithelial neoplasia in people living with HIV: a multicentre, randomised, non-inferiority trial (TECAIN-study) in the Springer journal "Infection"

Correspondence to: Stefan Esser, Clinic for Dermatology and Venerology, University Hospital Essen, University of Duisburg-Essen, Hufelandstr. 55, Essen 45122, Germany

stefan.esser@uk-essen.de; Tel. +49-201-723 3696

Table of contents

	Page
**METHODS**	
**List** of participating outpatient proctology/anal dysplasia units	2
**Case report form** for the documentation of the number, location, and extent of AIN-suspicious lesions	3
**RESULTS**	
**Supplementary Figure S1.** Outcome at 4-week follow-up (4WFU) and at 24-week follow-up (24WFU) according to smoking status at baseline, ITT and PP	4
**Supplementary Figure S2.** Outcome at 4-week follow-up (4WFU, top) and at 24-week follow-up (24WFU, bottom) according to CD4-cell count at baseline (< 350 vs. >= 350 CD4 cells/µL), ITT and PP	6
**Supplementary Table S1.** Adverse events (AE) in relation to the number of interventions in the period from baseline to four weeks after the end of treatment (4WFU) in the intention-to-treat (ITT) population	8
**Supplementary Table S2.** HPV detection and typing, HPV-DNA-load determination, and HPV-oncogene mRNA detection in TCA- and ECA-treated patients at baseline and at post-treatment weeks 4 (4WFU) and 24 (24WFU) in the intention-to-treat (**ITT**) population.	10
**Supplementary Table S3.** HPV detection and typing, HPV-DNA-load determination, and HPV-oncogene mRNA detection in TCA- and ECA-treated patients at baseline and at post-treatment weeks 4 (4WFU) and 24 (24WFU) in the per-protocol (**PP**) population.	13
**Supplementary Table S4.** Anal cytology results at baseline, at post-treatment weeks 4 (4WFU) and 24 (24WFU) in the intention-to-treat (ITT) population.	15

List of participating outpatient proctology/anal dysplasia units

- Department of Dermatology and Venereology, HPSTD (HIV/AIDS, Proctology, STD) Outpatient Clinic, University Hospital Essen, University Duisburg-Essen, Essen, Germany (hospital outpatient clinic; principal and clinical investigator: Prof S Esser MD)
- Department of Dermatology, Venereology and Allergology, Helios St. Elisabeth Hospital, Oberhausen, University Witten/Herdecke, Germany (hospital outpatient clinic; clinical investigator: Prof A Kreuter MD)
- Interdisciplinary Immunological Outpatient Clinic, Center for Sexual Health and Medicine, Department of Dermatology, Venereology and Allergology, Ruhr University Bochum, Bochum, Germany (hospital outpatient clinic; clinical investigator: Dr A Potthoff MD)
- Department of Infectious Diseases, Augustinerinnen Hospital, Cologne, Germany (hospital outpatient clinic; clinical investigator: Prof M Oette MD)
- Medical Practice, Drs Wiesel and de Vries, Berlin, Germany (specialist medical practice; clinical investigator: Dr P Wiesel MD)
- Department of Internal Medicine I, University Medical Center of the Johannes Gutenberg University Mainz, Mainz, Germany (hospital outpatient clinic; clinical investigator: Prof M Sprinzl MD)
- University Hospital Bonn, Department of General, Visceral, Thoracic and Vascular Surgery, Colorectal Surgery and Proctology, Bonn, Germany (hospital outpatient clinic; clinical investigator: Dr D Exner MD)

Case report form for the documentation of the number, location, and extent of AIN-suspicious lesions

Zentrum Nr.	Visiten-Datum: (Tag/Monat/Jahr)	Patienten-Nr.

(A7) Kartographie der Läsionen in Steinschnittlage (SSL)

High Resolution Anoskopie (HRA):

☐0 nein ☐1 ja

☞wenn nein, Begründung:

____

Gewebefärbung mit:

☐1 Essigsäure ☐2 Lugol`scher Lsg. ☐2 keine Färbung

☞wenn keine Färbung, Begründung

____

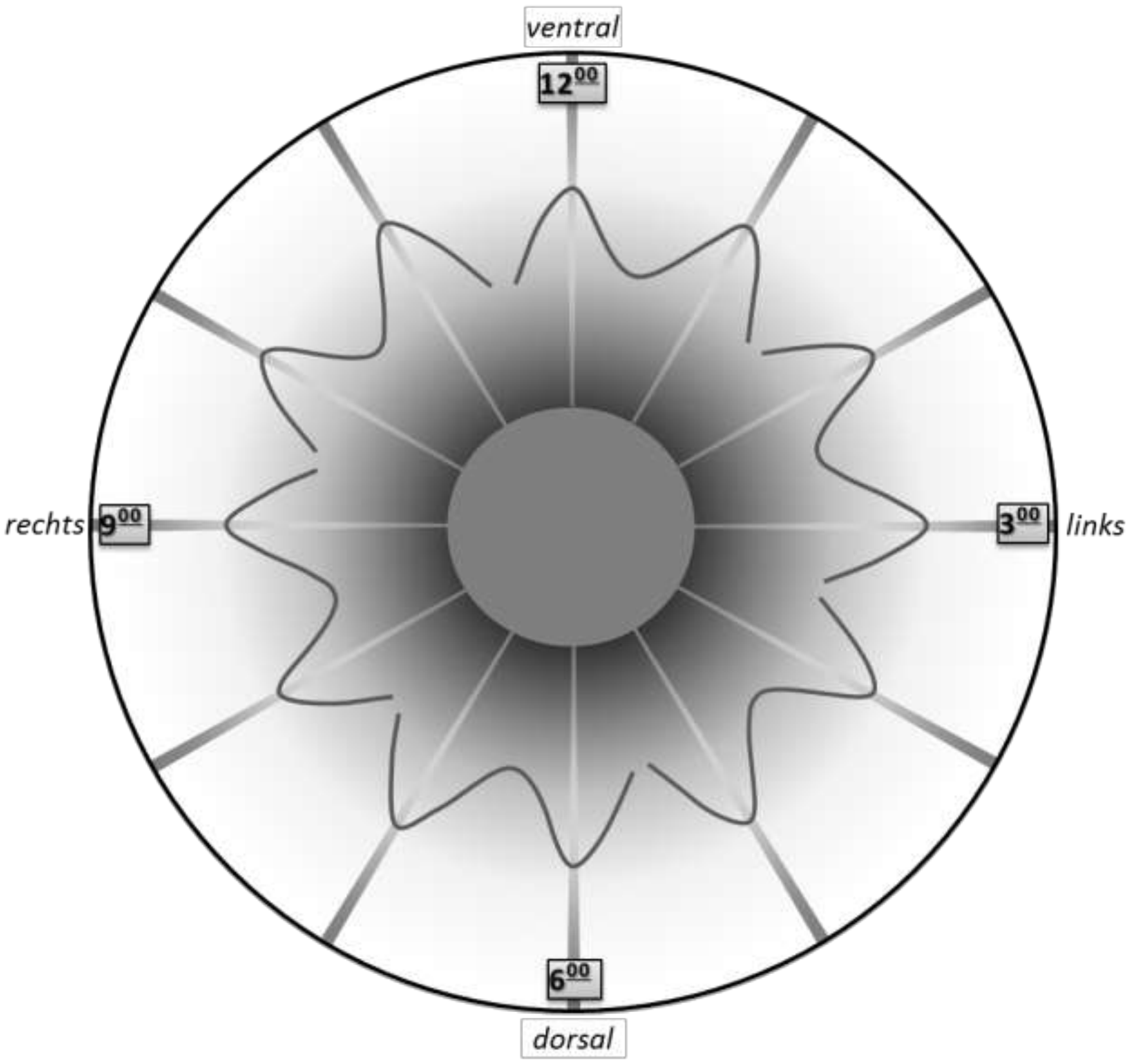

Datum _____ *Unterschrift Studienarzt* _____ *Name (Druckbuchstaben)*

**TECAIN**	Kartographien von HPV-assoziierten AIN-Läsionen<br>*Eine Anleitung zur Dokumentation befindet sich in der Studienbroschüre*	**A7**

Outcome 4WFU	Population	N TCA	N ECA	TCA %	ECA %	Difference in % [95% CI]
CCR and histological clearance/downgrade	ITT non-smokers	70	78	47.1	59.0	-11.8 [-27.8, 4.2]
CCR and histological clearance/downgrade	ITT current smokers	48	37	60.4	67.6	-7.2 [-27.6, 13.3]
Histological clearance/downgrade	ITT non-smokers	70	78	64.3	64.1	0.2 [-15.3, 15.7]
Histological clearance/downgrade	ITT current smokers	48	37	70.8	73.0	-2.1 [-21.4, 17.1]
CCR	ITT non-smokers	70	78	65.7	83.3	-17.6 [-31.5, -3.8]
CCR	ITT current smokers	48	37	75.0	81.1	-6.1 [-23.7, 11.5]
CCR and histological clearance/downgrade	PP non-smokers	53	69	58.5	65.2	-6.7 [-24.1, 10.7]
CCR and histological clearance/downgrade	PP current smokers	43	28	67.4	71.4	-4.0 [-25.8, 17.8]
Histological clearance/downgrade	PP non-smokers	53	69	73.6	71.0	2.6 [-13.4, 18.6]
Histological clearance/downgrade	PP current smokers	43	28	79.1	78.6	0.5 [-19.0, 20.0]
CCR	PP non-smokers	53	69	75.5	87.0	-11.5 [-25.5, 2.6]
CCR	PP current smokers	43	28	83.7	85.7	-2.0 [-19.0, 15.0]

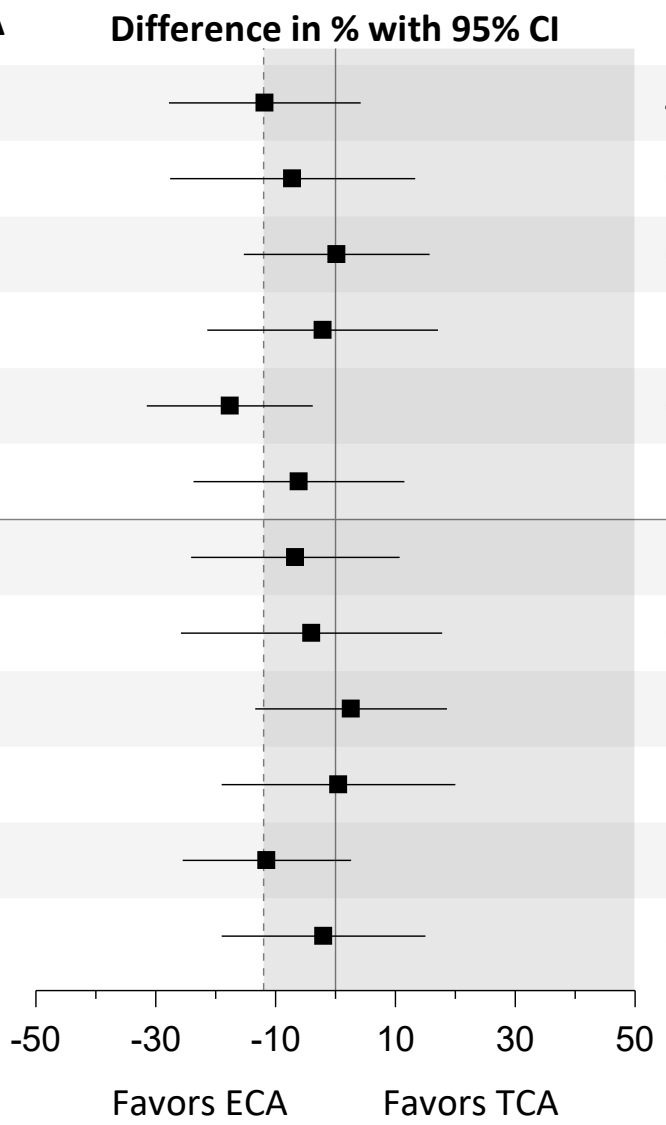

Outcome 24WFU	Population	N TCA	N ECA	TCA %	ECA %	Difference in % [95% CI]
CCR and histological clearance/downgrade	ITT non-smokers	70	78	51.4	48.7	2.7 [-13.4, 18.8]
CCR and histological clearance/downgrade	ITT current smokers	48	37	50.0	48.6	1.4 [-20.1, 22.8]
Histological clearance/downgrade	ITT non-smokers	70	78	60.0	64.1	-4.1 [-19.8, 11.6]
Histological clearance/downgrade	ITT current smokers	48	37	58.3	54.1	4.3 [-17.0, 25.5]
CCR	ITT non-smokers	70	78	71.4	67.9	3.5 [-11.3, 18.3]
CCR	ITT current smokers	48	37	79.2	73.0	6.2 [-12.2, 24.5]
CCR and histological clearance/downgrade	PP non-smokers	53	69	64.2	50.7	13.4 [-4.1, 30.9]
CCR and histological clearance/downgrade	PP current smokers	43	28	55.8	60.7	-4.9 [-28.3, 18.5]
Histological clearance/downgrade	PP non-smokers	53	69	69.8	68.1	1.7 [-14.8, 18.2]
Histological clearance/downgrade	PP current smokers	43	28	62.8	67.9	-5.1 [-27.6, 17.5]
CCR	PP non-smokers	53	69	83.0	68.1	14.9 [0.0, 29.8]
CCR	PP current smokers	43	28	86.0	75.0	11.0 [-8.0, 30.1]

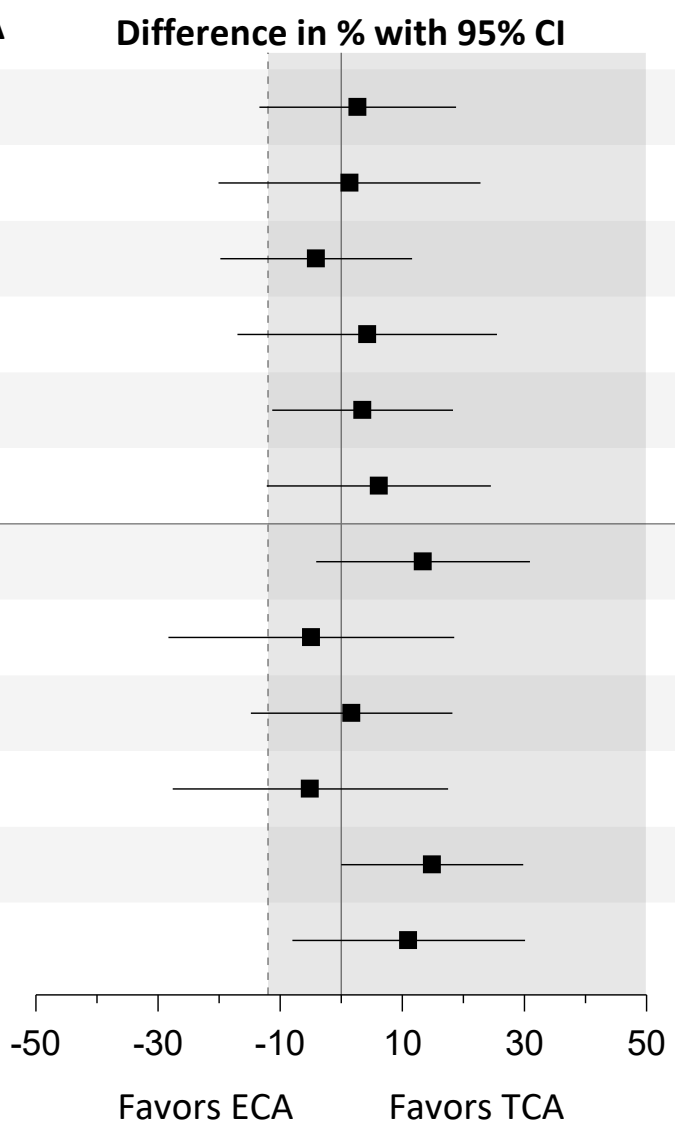

Supplementary Figure S1 Outcome at 4-weeks-follow-up (4WFU, top) and at 24-weeks-follow-up (24WFU, bottom) according to smoking status at baseline, ITT and PP

Forest plots of the absolute differences in % of the two treatment groups TCA and ECA at 4WFU (top panel) and at 24WFU (bottom panel), differentiating between non-smokers (dark grey background) and current smokers (light grey background) at baseline, for the ITT (top) and PP (bottom) population, respectively. Non-smokers comprise ex-smokers and patients who never smoked. The following three outcomes are shown from top to bottom, respectively: 1.) complete clinical response (CCR) and histological clearance/downgrade, 2.) histological clearance/downgrade, and 3.) CCR.

The vertical dashed black line and the grey area right of the line represent the non-inferiority margin and the area above the margin of -12%. The displayed differences in % are based on the exact differences rounded after subtraction. Because of rounding, the displayed differences can differ slightly (+/- 0.1%) from the differences of the shown TCA minus ECA response rates.

CI, confidence interval; CCR, complete clinical response; ECA, electrocautery; ITT, intention-to-treat; N, number of patients; PP, per-protocol; TCA, trichloroacetic acid; 4WFU, 4-weeks-follow-up; 24WFU, 24-weeks-follow-up.

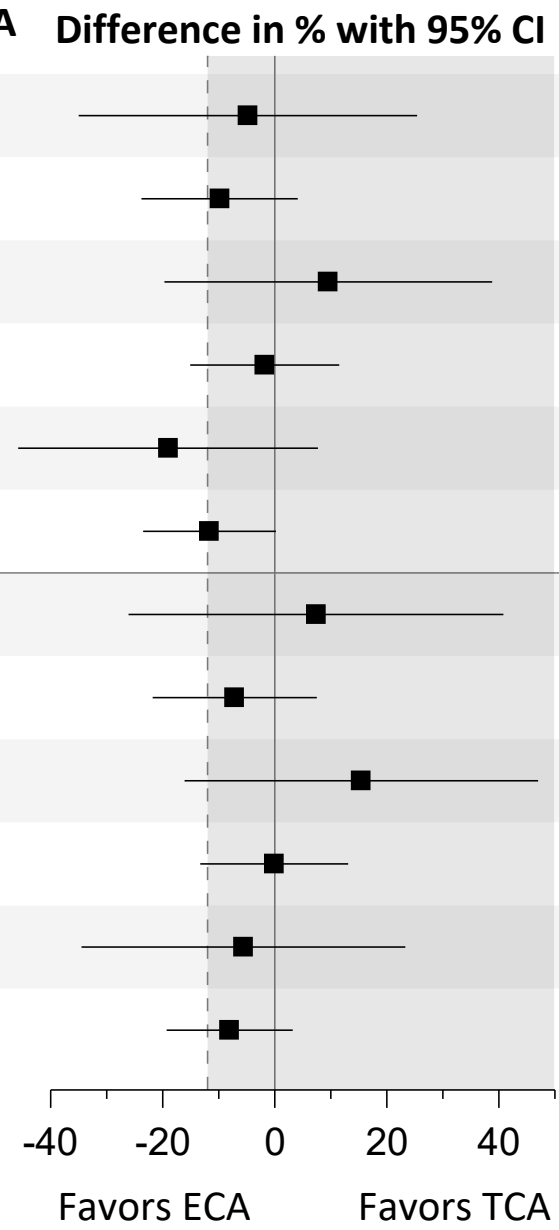

Outcome 4WFU	Population	N TCA	N ECA	TCA %	ECA %	Difference in % [95% CI]
CCR and histological clearance/downgrade	ITT CD4 <350	21	21	47.6	52.4	-4.8 [-35.0, 25.4]
CCR and histological clearance/downgrade	ITT CD4 >=350	97	93	53.6	63.4	-9.8 [-23.8, 4.1]
Histological clearance/downgrade	ITT CD4 <350	21	21	66.7	57.1	9.5 [-19.7, 38.8]
Histological clearance/downgrade	ITT CD4 >=350	97	93	67.0	68.8	-1.8 [-15.1, 11.5]
CCR	ITT CD4 <350	21	21	61.9	81.0	-19.0 [-45.8, 7.7]
CCR	ITT CD4 >=350	97	93	71.1	82.8	-11.7 [-23.5, 0.2]
CCR and histological clearance/downgrade	PP CD4 <350	15	19	60.0	52.6	7.4 [-26.1, 40.8]
CCR and histological clearance/downgrade	PP CD4 >=350	81	77	63.0	70.1	-7.2 [-21.8, 7.5]
Histological clearance/downgrade	PP CD4 <350	15	19	73.3	57.9	15.4 [-16.1, 47.0]
Histological clearance/downgrade	PP CD4 >=350	81	77	76.5	76.6	-0.1 [-13.3, 13.1]
CCR	PP CD4 <350	15	19	73.3	78.9	-5.6 [-34.5, 23.3]
CCR	PP CD4 >=350	81	77	80.2	88.3	-8.1 [-19.3, 3.2]

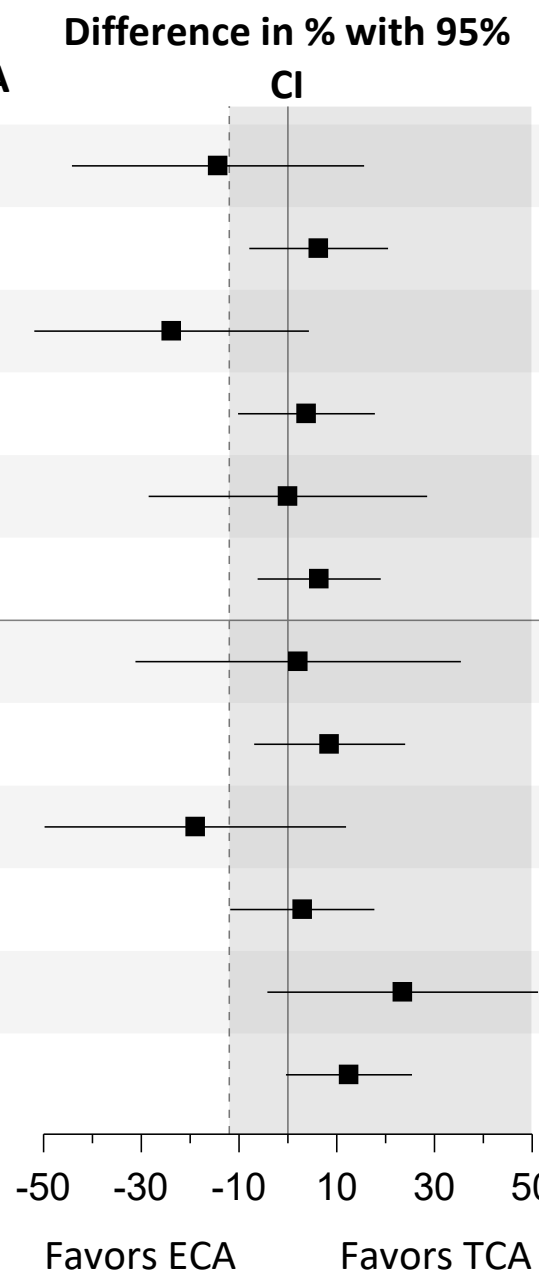

Outcome 24WFU	Population	N TCA	N ECA	TCA %	ECA %	Difference in % [95% CI]
CCR and histological clearance/downgrade	ITT CD4 <350	21	21	42.9	57.1	-14.3 [-44.2, 15.6]
CCR and histological clearance/downgrade	ITT CD4 >=350	97	93	52.6	46.2	6.3 [-7.9, 20.5]
Histological clearance/downgrade	ITT CD4 <350	21	21	52.4	76.2	-23.8 [-51.9, 4.3]
Histological clearance/downgrade	ITT CD4 >=350	97	93	60.8	57.0	3.8 [-10.2, 17.8]
CCR	ITT CD4 <350	21	21	66.7	66.7	0.0 [-28.5, 28.5]
CCR	ITT CD4 >=350	97	93	76.3	69.9	6.4 [-6.2, 19.0]
CCR and histological clearance/downgrade	PP CD4 <350	15	19	60.0	57.9	2.1 [-31.2, 35.4]
CCR and histological clearance/downgrade	PP CD4 >=350	81	77	60.5	51.9	8.5 [-6.9, 24.0]
Histological clearance/downgrade	PP CD4 <350	15	19	60.0	78.9	-18.9 [-49.8, 11.9]
Histological clearance/downgrade	PP CD4 >=350	81	77	67.9	64.9	3.0 [-11.8, 17.7]
CCR	PP CD4 <350	15	19	86.7	63.2	23.5 [-4.2, 51.2]
CCR	PP CD4 >=350	81	77	84.0	71.4	12.5 [-0.4, 25.4]

Supplementary Figure S2 Outcome at 4-weeks-follow-up (4WFU, top) and at 24-weeks-follow-up (24WFU, bottom) according to CD4-cell count at baseline (< 350 vs. >= 350 CD4 cells/µL), ITT and PP

Forest plots of the absolute differences in % of the two treatment groups TCA and ECA at 4WFU (top panel) and at 24WFU (bottom panel), differentiating between patients with CD4-cell counts below (dark grey background) and above (light grey background) 350 cells/µL at baseline, for the ITT (top) and PP (bottom) population, respectively. The following three outcomes are shown from top to bottom,

respectively: 1.) complete clinical response (CCR) and histological clearance/downgrade, 2.) histological clearance/downgrade, and 3.) CCR.

The vertical dashed black line and the grey area right of the line represent the non-inferiority margin and the area above the margin of -12%. The displayed differences in % are based on the exact differences rounded after subtraction. Because of rounding, the displayed differences can differ slightly (+/- 0.1%) from the differences of the shown TCA minus ECA response rates.

CI, confidence interval; CCR, complete clinical response; ECA, electrocautery; ITT, intention-to-treat; N, number of patients; PP, per-protocol; TCA, trichloroacetic acid; 4WFU, 4-weeks-follow-up; 24WFU, 24-weeks-follow-up.

Supplementary Table S1 Adverse events (AE) in relation to the number of interventions in the period from baseline to four weeks after the end of treatment (4WFU) in the intention-to-treat (ITT) population

	**TCA** (n=324)	**ECA** (n=265)	**Difference of AE percentage values** (95% CI)
**Any AE**, highest grade	156 (48.1%)	138 (52.1%)	-3.9 (-12.0 to 4.2)
none	168 (51.9%)	127 (47.9%)	3.9 (-4.2 to 12.0)
Grade 1 (mild)	95 (29.3%)	62 (23.4%)	5.9 (-1.2 to 13.0)
Grade 2 (moderate)	48 (14.8%)	55 (20.8%)	-5.9 (-12.2 to 0.2)
Grade 3 (severe)	13 (**4.0**%)	21 (**7.9**%)	**-3.9** (-7.8 to -0.0)
**Treatment-related AE**,[a] highest grade	141 (43.5%)	132 (49.8%)	-6.3 (-14.4 to 1.8)
**Pain**	68 (**21.0**%)	96 (**36.2**%)	**-15.2** (-22.5 to -7.9)
none	256 (**79.0**%)	169 (**63.8**%)	**15.2** (7.9 to 22.6)
Grade 1 (mild)	43 (13.3%)	46 (17.4%)	-4.1 (-10.0 to 1.8)
Grade 2 (moderate)	20 (**6.2**%)	32 (**12.1**%)	**-5.9** (-10.6 to -1.2)
Grade 3 (severe)	5 (**1.5**%)	18 (**6.8**%)	**-5.2** (-8.6 to -1.9)
**Burning**	59 (18.2%)	39 (14.7%)	3.5 (-2.5 to 9.5)
none	265 (81.8%)	226 (85.3%)	-3.5 (-9.5 to 2.5)
Grade 1 (mild)	45 (**13.9**%)	18 (**6.8**%)	**7.1** (2.3 to 11.9)
Grade 2 (moderate)	12 (3.7%)	16 (6.0%)	-2.3 (-5.9 to 1.2)
Grade 3 (severe)	2 (0.6%)	5 (1.9%)	-1.3 (-3.1 to 0.6)
**Itching**	26 (8.0%)	14 (5.3%)	2.7 (-1.3 to 6.7)
none	298 (92.0%)	251 (94.7%)	-2.7 (-6.7 to 1.3)
Grade 1 (mild)	22 (6.8%)	12 (4.5%)	2.3 (-1.4 to 6.0)
Grade 2 (moderate)	4 (1.2%)	1 (0.4%)	0.9 (-0.6 to 2.3)
Grade 3 (severe)	0 (0.0%)	1 (0.4%)	-0.4 (-1.1 to 0.4)
**Spasms**	8 (2.5%)	4 (1.5%)	1.0 (-1.3 to 3.2)
none	316 (97.5%)	261 (98.5%)	-1.0 (-3.2 to 1.3)
Grade 1 (mild)	5 (1.5%)	1 (0.4%)	1.2 (-0.4 to 2.7)
Grade 2 (moderate)	2 (0.6%)	2 (0.8%)	-0.1 (-1.5 to 1.2)
Grade 3 (severe)	1 (0.3%)	1 (0.4%)	-0.1 (-1.0 to 0.9)
**Irregular bowel movements**[b]	52 (16.0%)	39 (14.7%)	1.3 (-4.5 to 7.2)
none	272 (84.0%)	226 (85.3%)	-1.3 (-7.2 to 4.5)
Grade 1 (mild)	44 (13.6%)	35 (13.2%)	0.4 (-5.2 to 5.9)
Grade 2 (moderate)	5 (1.5%)	4 (1.5%)	0.0 (-2.0 to 2.0)
Grade 3 (severe)	3 (0.9%)	0 (0.0%)	0.9 (-0.1 to 2.0)
**Bleeding**[c]	8 (2.5%)	11 (4.2%)	-1.7 (-4.6 to 1.3)
none	316 (97.5%)	254 (95.8%)	1.7 (-1.3 to 4.6)
Grade 1 (mild)	6 (1.9%)	5 (1.9%)	0.0 (-2.2 to 2.2)
Grade 2 (moderate)	2 (0.6%)	6 (2.3%)	-1.6 (-3.7 to 0.3)
Grade 3 (severe)	0 (0.0%)	0 (0.0%)	0.0
**Other treatment-related AE**[d]	0 (0.0%)	3 (1.1%)	-1.1 (-2.4 to 0.1)
**Median number of treatment-related AE per intervention** (IQR)	0 (0 - 1)	1 (0 – 1)	**p=0.02** [e]
**Non-treatment-related AE**	30 (9.3%)	20 (7.5%)	1.7 (-2.8 to 6.2)
**SAE**[f]	4 (1.2%)	4 (1.5%)	-0.3 (-2.2 to 1.6)
**Treatment-related SAE**	0 (0.0%)	0 (0.0%)	0.0
**Study discontinuation due to SAE**[f]	0 (0.0%)	1 (0.4%)	-0.4 (-1.1 to 0.4)
**Anaesthesia use**	28 (**8.6**%)	105 (**39.6**%)	**-31.0** (-37.6 to -24.3)
none	296 (**91.4**%)	160 (**60.4**%)	**31.0** (24.3 to 37.6)
local	10 (**3.1**%)	79 (**29.8**%)	**-26.7** (-32.5 to -20.9)
systemic sedatives	18 (5.6%)	25 (9.4%)	-3.9 (-8.2 to 0.4)
general anaesthesia[g]	0 (0.0%)	1 (0.4%)	-0.4 (-1.1 to 0.4)

AE, adverse event; ECA, electrocautery; IQR, interquartile range; SAE, serious adverse event; TCA, trichloroacetic acid.

Unless otherwise stated data are in n (%). Percentage figures for AE that were significantly different between the TCA- and the ECA-group are printed in bold. Percentages may not total 100 due to rounding.

324 interventions were performed in the TCA group (118 patients at baseline) and 265 interventions in the ECA group (115 patients at baseline) until the end of treatment. 4WFU data was available for 221 patients (109 for TCA, 112 for ECA, see Table 2).

[a] Treatment-related self-reported AE include pain, burning, itching, spasms, irregular bowel movements, and bleeding. Physician-reported AE include bleeding, wound healing disorder, wound infection, scarring, fissure formation, anal stenosis, or allergic reactions. In terms of severity, the highest grade reported up to 4WFU was taken into account.

[b] Irregular bowel movements comprise obstipation, rectal discharge, faecal deposits, thin faeces, urge to defecate, diarrhoea and flatulence.

[c] Bleeding comprises both bleeding reported by the patient and bleeding during or directly after treatment reported by the physician.

[d] Other treatment-related AE in two patients of the ECA-group were post-operative wound healing disorder (grade 2) and fissure formation after the last treatment (grade 3) in one patient and a post-operative wound infection after the last treatment in another patient (grade 2). Scar formation, anal stenosis, or allergic reactions did not occur in any patient.

[e] Wilcoxon test

[f] The four SAE in the TCA-group were recurrent herniated intervertebral discs, ear surgery, benign prostate hyperplasia, and hospitalisation for surgical removal of anogenital warts four weeks after AIN-treatment. The four SAE in the ECA-group were angina pectoris (four weeks after the end of treatment), an apoplectic stroke (three weeks after the end of treatment), multi-substance abuse, and hospitalisation for surgical removal of expanding intraanal AIN. The patient with the latter SAE discontinued the study due to hospitalisation for surgical removal of the expanding AIN three weeks after the baseline visit. The eight SAE mentioned occurred in one patient each and all of them were not considered treatment-related.

[g] General anaesthesia was only necessary for one ECA intervention (and was never applied in the TCA group). Systemic sedatives included oral or intravenous medication for sedation. Oral or intravenous medication for sedation was given to five patients of the TCA-group for 18 interventions and to seven patients of the ECA-group for 25 interventions. Local anaesthesia was given to five patients of the TCA-group for 10 interventions and to 32 patients of the ECA-group for 79 interventions. Both local anaesthesia and systemic sedatives were used for three interventions in the TCA- and for four interventions in the ECA-group.

Supplementary Table S2 HPV detection and typing, HPV-DNA-load determination, and HPV-oncogene mRNA detection in TCA- and ECA-treated patients at baseline and at post-treatment weeks 4 (4WFU) and 24 (24WFU) in the intention-to-treat (ITT) population

	TCA n=118				ECA n=115				
**ITT**	**Baseline, before treatment**	**4WFU**	**24WFU**	**p-values* TCA** a. 4WFU vs. baseline b. 24WFU vs. baseline	**Baseline, before treatment**	**4WFU**	**24WFU**	**p-values* ECA** a. 4WFU vs. baseline b. 24WFU vs. baseline	**p-values* TCA vs. ECA** a.baseline b. 4WFU c. 24WFU
**Evaluated Samples** (Samples missing or not evaluable or patients lost to follow-up)	116 (2)	106 (12)	104 (14)		112 (3)	108 (7)	102 (13)		
**HPV prevalence** N (%) [95% CI]									
Any HPV-type[a]	111 (95.7%) [90.2 to 98.6]	101 (95.3%) [89.3 to 98.5]	96 (92.3%) [85.4 to 96.6]	a. p=0.32 b. p=0.26	109 (97.3%) [92.4 to 99.4]	104 (96.3%) [90.8 to 99.0]	97 (95.1%) [88.9 to 98.4]	a. p=0.56 b. p=0.56	a. p=0.50 b. p=0.71 c. p=0.41
HR-HPV-types[b]	93 (80.2%) [71.7 to 87.0]	83 (78.3%) [69.2 to 85.7]	77 (74.0%) [64.5 to 82.1]	a. p=1.00 b. p=0.25	89 (79.5%) [70.8 to 86.5]	89 (82.4%) [73.9 to 89.1]	84 (82.4%) [73.6 to 89.2]	a. p=0.20 b. p=0.25	a. p=0.89 b. p=0.44 c. p=0.41
pHR-HPV-types[c]	38 (32.8%) [24.3 to 42.1]	42 (39.6%) [30.3 to 49.6]	27 (26.0%) [17.9 to 35.5]	a. p=0.26 b. p=0.09	22 (19.6%) [12.7 to 28.2]	31 (28.7%) [20.4 to 38.2]	26 (25.5%) [17.4 to 35.1]	a. p=**0.04** b. p=0.35	a. p=**0.03** b. p=0.09 c. p=0.94
HR- and pHR-HPV-types[b,c]	101 (87.1%) [79.6 to 92.6]	90 (84.9%) [76.7 to 91.1]	83 (79.8%) [70.8 to 87.0]	a. p=0.78 b. p=0.06	95 (84.8%) [76.8 to 90.9]	95 (88.0%) [80.3 to 93.4]	89 (87.3%) [79.2 to 93.0]	a. p=0.25 b. p=0.32	a. p=0.62 b. p=0.51 c. p=0.15
LR-HPV-types[d] (samples with and without HR/pHR-types)	78 (67.2%) [57.9 to 75.7]	65 (61.3%) [51.4 to 70.6]	64 (61.5%) [51.5 to 70.9]	a. p=0.41 b. p=0.26	78 (69.6%) [60.2 to 78.0]	62 (57.4%) [47.5 to 66.9]	54 (52.9%) [42.8 to 62.9]	a. p=**0.02** b. p=**0.01**	a. p=0.26 b. p=0.45 c. p=0.15
Only LR-HPV-types[d] (samples without HR/pHR-types)	10 (8.6%) [4.2 to 15.3]	11 (10.4%) [5.3 to 17.8]	13 (12.5%) [6.8 to 20.4]	a. p=0.56 b. p=0.20	14 (12.5%) [7.0 to 20.1]	9 (8.3%) [3.9 to 15.2]	8 (7.8%) [3.4 to 14.9]	a. p=0.13 b. p=0.17	a. p=0.34 b. p=0.61 c. p=0.15
HPV16	44 (37.9%) [29.1 to 47.4]	44 (41.5%) [32.0 to 51.5]	39 (37.5%) [28.2 to 47.5]	a. p=0.83 b. p=1.00	49 (43.8%) [34.4 to 53.4]	47 (43.5%) [34.0 to 53.4]	43 (42.2%) [32.4 to 52.3]	a. p=0.78 b. p=0.62	a. p=0.37 b. p=0.77 c. p=0.49
HPV16/18/31/33/45/52/58[e]	82 (70.7%) [61.5 to 78.8]	67 (63.2%) [53.3 to 72.4]	67 (64.4%) [54.4 to 73.6]	a. p=0.22 b. p=0.24	82 (73.2%) [64.0 to 81.1]	79 (73.1%) [63.8 to 81.2]	77 (75.5%) [66.0 to 83.5]	a. p=0.56 b. p=0.35	a. p=0.67 b. p=0.12 c. p=0.08
**HPV multiplicity** Median [IQR], max.									
No. of HPV-types[a]	3 [2 to 3], 12	3 [1 to 4], 6	2 [1 to 3.5], 7	a. p=0.38 b. p=**0.02**	2 [1.5 to 4], 12	2 [1 to 4], 10	2 [1 to 3], 7	a. p=0.63 b. p=0.29	a. p=0.40 b. p=0.38 c. p=0.87

No. of HR-HPV-types[b]	1 [1 to 2], 7	1 [1 to 2], 5	1 [0 to 2], 4	a. p=**0.01**<br>b. p=**0.02**	1 [1 to 2], 6	1 [1 to 2], 4	1 [1 to 2], 5	a. p=**0.01**<br>b. p=**0.01**	a. p=0.89<br>b. p=0.45<br>c. p=0.49
No. of pHR-HPV-types[c]	0 [0 to 1], 3	0 [0 to 1], 2	0 [0 to 1], 3	a. p=0.65<br>b. p=0.07	0 [0 to 0], 4	0 [0 to 1], 4	0 [0 to 1], 4	a. p=0.18<br>b. p=0.53	a. p=**0.02**<br>b. p=0.10<br>c. p=0.92
No. of HR- and pHR-HPV-types[b,c]	1 [1 to 3], 10	2 [1 to 3], 6	1 [1 to 2], 5	a. p=0.77<br>b. p=0.06	1 [1 to 2], 7	2 [1 to 3], 7	1.5 [1 to 2], 6	a. p=0.19<br>b. p=0.77	a. p=0.46<br>b. p=0.58<br>c. p=0.49
No. of LR-HPV-types[d] (in samples with and without HR/pHR-types)	1 [0 to 2], 3	1 [0 to 1], 3	1 [0 to 1], 3	a. p=**0.02**<br>b. p=**0.02**	1 [0 to 2], 5	1 [0 to 1], 5	1 [0 to 1], 4	a. p=**0.01**<br>b. p=**0.01**	a. p=0.76<br>b. p=0.81<br>c. p=0.51
No. of LR-HPV-types[d] (in samples without HR/pHR-types)	1.5 [1 to 2], 3	1 [1 to 1], 2	1 [1 to 1], 3	a. p=0.50<br>b. p=1.00	1 [1 to 2], 2	1 [1 to 1], 2	1 [1 to 2], 3	a. p=1.00<br>b. p=1.00	a. p=1.00<br>b. p=1.00<br>c. p=1.00
No. of HPV16/18/31/33/45/52/58[e]	1 [0 to 2], 5	1 [0 to 2], 4	1 [0 to 2], 4	a. p=0.67<br>b. p=0.28	2 [0 to 2], 5	2 [0 to 2], 4	2 [1 to 2], 5	a. p=0.77<br>b. p=0.66	a. p=0.45<br>b. p=0.55<br>c. p=0.11
**HPV-oncogene-mRNA detection**[f]<br>N (%) [95% CI]	90 (77.6%)<br>[68.9 to 84.8]	81 (76.4%)<br>[67.2 to 84.1]	81 (77.9%)<br>[68.7 to 85.4]	a. p=1.00<br>b. p=0.83	86 (76.8%)<br>[67.9 to 84.2]	81 (75.0%)<br>[65.7 to 82.8]	75 (73.5%)<br>[63.9 to 81.8]	a. p=0.83<br>b. p=0.65	a. p=0.88<br>b. p=0.81<br>c. p=0.30
**HPV-DNA loads**[g]<br>Median [IQR], n									
HPV16-DNA load	11.6 [0.6 to 98.9], n=44	11.5 [0.5 to 87.7], n=44	12.7 [1.0 to 64.3], n=38	a. p=0.94<br>b. p=0.60	6.7 [0.1 to 32.9], n=48	2.4 [0.2 to 35.8], n=45	2.8 [0.2 to 101.7], n=40	a. p=0.42<br>b. p=0.37	a. p=0.13<br>b. p=0.09<br>c. p=0.20
HPV16+18-DNA load	12.1 [0.7 to 96.7], n=11	3.0 [0.4 to 97.5], n=9	11.6 [1.1 to 53.3], n=11	a. p=0.47<br>b. p=0.88	6.7 [0.1 to 32.9], n=12	2.7 [0.2 to 34.6], n=9	2.4 [0.2 to 38.1], n=11	a. p=0.51<br>b. p=0.54	a. p=0.08<br>b. p=0.11<br>c. p=0.11
HR-HPV-DNA load[h]	15.5 [0.9 to 178.8], n=86	14.3 [2.4 to 137.8], n=75	17.4 [1.1 to 80.9], n=71	a. p=0.33<br>b. p=0.98	20.4 [0.9 to 99.6], n=83	6.2 [0.7 to 54.6], n=81	5.6 [0.5 to 93.5], n=75	a. p=**0.04**<br>b. p=0.80	a. p=0.51<br>b. p=0.11<br>c. p=0.11

4WFU, 4-weeks-follow-up (4 weeks after the end of treatment); 24WFU, 24-weeks-follow-up (24 weeks after the end of treatment); CI, confidence interval; ECA, electrocautery; HPV, Human papillomavirus; HR, high-risk; IQR, interquartile range; ITT, intention-to-treat; LR, low-risk; max, maximum; PP, per-protocol; TCA, trichloroacetic acid.

[a] Any HPV-types comprises 41 HPV-types analysed in all samples by bead-based hybridization: HPV6, 11, 16, 18, 26, 30, 31, 33, 34, 35, 39, 40, 42, 43, 44, 45, 51, 52, 53, 54, 55, 56, 57, 58, 59, 61, 66, 67, 68, 69, 70, 71, 72, 73, 81, 82, 83, 84, 85, 89, and 97. In seven HPV-positive anal swabs none of the 41 HPV-types included in bead-based hybridization could be detected. Sequencing of the respective PCR-products revealed HPV27 (n=1), HPV74 (n=1), HPV78 (n=1), and HPV91 (n=3) in six samples. In one HPV-positive swab no HPV-type could be determined (not typifiable).

[b] 13 high-risk (HR) HPV-types: HPV16, 18, 31, 33, 35, 39, 45, 51, 52, 56, 58, 59, 68

[c] 12 possible high-risk (pHR) HPV-types: HPV 26, 30, 34, 53, 66, 67, 69, 70, 73, 82, 85, 97

[d] 16 low-risk (LR) HPV-types: HPV6, 11, 40, 42, 43, 44, 54, 55, 57, 61, 71, 72, 81, 83, 84, 89

[e] HPV16/18/31/33/45/52/58 are the HR-HPV-types included in the nonavalent HPV vaccine.

[f] HPV-oncogene-mRNA detection comprises the qualitative detection of viral oncogene (E6/E7) messenger RNA (mRNA) from 13 HR-HPV-types (HPV16, 18, 31, 33, 35, 39, 45, 51, 52, 56, 58, 59, 68) and 1 pHR-HPV-type (HPV66). The assay (Aptima HPV assay, Hologic) does not discriminate between the 14 HPV-types included.

[g] Type-specific HPV-DNA loads were only determined in samples that were positive for one or more of the following ten HR-HPV-types: HPV16, 18, 31, 33, 45, 51, 52, 56, 58, 59. Type-specific HPV-DNA loads are expressed as HPV-DNA copies per beta-globin gene copy, e.g., HPV16-DNA load as HPV16-DNA copies per beta-globin-gene copy. Cumulative HPV-DNA loads were calculated if two or more of the 10 quantified HR-HPV-types were present in a sample.

[h] HR-HPV-DNA load is HPV16 + HPV18 + HPV31 + HPV33 + HPV45 + HPV51 + HPV52 + HPV56 + HPV58 + HPV59 cumulative DNA load (if the respective HPV-types were present).

[*] p-values < 0.05 are printed in bold. Changes were calculated per patient. McNemar’s test (binary outcome) and Wilcoxon signed-rank test (HPV multiplicity and HPV-DNA loads) were used for paired samples, i.e. comparisons to baseline for each treatment group separately. Fisher’s exact test (binary outcome) and two-sided Mann-Whitney U test were performed for unpaired samples, i.e. comparing the two treatment groups.

Supplementary Table S3 HPV detection and typing, HPV-DNA-load determination, and HPV-oncogene mRNA detection in TCA- and ECA-treated patients at baseline and at post-treatment weeks 4 (4WFU) and 24 (24WFU) in the per-protocol (PP) population

	**TCA n=96**				**ECA n=97**				
**PP**	**Baseline, before treatment**	**4WFU**	**24WFU**	**p-values* TCA** a. 4WFU vs. baseline b. 24WFU vs. baseline	**Baseline, before treatment**	**4WFU**	**24WFU**	**p-values* ECA** a. 4WFU vs. baseline b. 24WFU vs. baseline	**p-values* TCA vs. ECA** a.baseline b. 4WFU c. 24WFU
**Evaluated Samples** (Samples missing or not evaluable or patients lost to follow-up)	94 (2)	95 (1)	92 (4)		95 (2)	95 (2)	90 (7)		
**HPV prevalence** N (%) [95% CI]									
Any HPV-type[a]	90 (95.7%) [89.5 to 98.8]	91 (95.8%) [89.6 to 98.8]	85 (92.4%) [84.9 to 96.9]	a. p=0.32 b. p=0.41	92 (96.8%) [91.0 to 99.3]	91 (95.8%) [89.6 to 98.8]	85 (94.4%) [87.5 to 98.2]	a. p=0.56 b. p=0.56	a. p=0.72 b. p=1.00 c. p=0.77
HR-HPV-types[b]	73 (77.7%) [67.9 to 85.6]	75 (78.9%) [69.4 to 86.6]	68 (73.9%) [63.7 to 82.5]	a. p=0.56 b. p=0.62	75 (78.9%) [69.4 to 86.6]	79 (83.2%) [74.1 to 90.1]	74 (82.2%) [72.7 to 89.5]	a. p=0.20 b. p=0.35	a. p=0.86 b. p=0.58 c. p=0.21
pHR-HPV-types[c]	31 (33.0%) [23.6 to 43.0]	39 (41.1%) [31.1 to 51.6]	23 (25.0%) [16.6 to 35.1]	a. p=0.14 b. p=0.13	18 (18.9%) [11.6 to 28.3]	25 (26.3%) [17.8 to 36.4]	21 (23.3%) [15.1 to 33.4]	a. p=0.13 b. p=0.44	a. p=**0.03** b. p=0.05 c. p=0.86
HR- and pHR-HPV-types[b,c]	81 (86.2%) [77.5 to 92.4]	82 (86.3%) [77.7 to 92.5]	74 (80.4%) [70.9 to 88.0]	a. p=0.76 b. p=0.20	81 (85.3%) [76.5 to 91.7]	85 (89.5%) [81.5 to 94.8]	78 (86.7%) [77.9 to 92.9]	a. p=0.25 b. p=0.59	a. p=1.00 b. p=0.66 c. p=0.32
LR-HPV-types[d] (samples with and without HR/pHR-types)	60 (63.8%) [53.3 to 73.5]	55 (57.9%) [47.3 to 68.0]	54 (58.7%) [47.9 to 68.9]	a. p=0.41 b. p=0.43	64 (67.4%) [57.0 to 76.6]	52 (54.7%) [44.2 to 65.0]	45 (50.0%) [39.3 to 60.7]	a. p=**0.02** b. p=**0.01**	a. p=0.65 b. p=0.77 c. p=0.30
Only LR-HPV-types[d] (samples without HR/pHR-types)	9 (9.6%) [4.5 to 17.4]	9 (9.5%) [4.4 to 17.2]	11 (12.0%) [6.1 to 20.4]	a. p=1.00 b. p=0.41	11 (11.6%) [5.9 to 19.8]	6 (6.3%) [2.4 to 13.2]	7 (7.8%) [3.2 to 15.4]	a. p=0.13 b. p=0.37	a. p=0.81 b. p=0.59 c. p=0.46
HPV16	35 (37.2%) [27.5 to 47.8]	43 (45.3%) [35.0 to 55.8]	36 (39.1%) [29.1 to 49.9]	a. p=**0.01** b. p=0.56	41 (43.2%) [33.0 to 53.7]	43 (45.3%) [35.0 to 55.8]	39 (43.3%) [32.9 to 54.2]	a. p=0.56 b. p=0.44	a. p=0.41 b. p=1.00 c. p=0.65
HPV16/18/31/33/45/52/58[e]	63 (67.0%) [56.6 to 76.4]	67 (70.5%) [60.3 to 79.4]	62 (67.4%) [56.8 to 77.8]	a. p=0.81 b. p=1.00	68 (71.6%) [61.4 to 80.4]	79 (83.2%) [74.1 to 90.1]	69 (76.7%) [66.6 to 84.9]	a. p=0.56 b. p=0.47	a. p=0.53 b. p=0.35 c. p=0.26
**HPV multiplicity** Median [IQR], max.									
No. of HPV-types[a]	2 [1 to 4], 7	3 [1 to 4], 6	2 [1 to 3], 7	a. p=0.96 b. p=0.13	2 [1 to 4], 12	2 [1 to 3], 10	2 [1 to 3], 7	a. p=0.41 b. p=0.27	a. p=0.77 b. p=0.20 c. p=0.92

No. of HR-HPV-types[b]	1 [1 to 2], 5	1 [1 to 2], 5	1 [0 to 2], 4	a. p=**0.04**<br>b. p=0.20	1 [1 to 2], 6	1 [1 to 2], 4	1 [1 to 2], 5	a. p=**0.01**<br>b. p=**0.01**	a. p=0.83<br>b. p=0.46<br>c. p=0.18
No. of pHR-HPV-types[c]	0 [0 to 1], 2	0 [0 to 1], 2	0 [0 to 0.5], 3	a. p=0.26<br>b. p=0.13	0 [0 to 0], 4	0 [0 to 1], 4	0 [0 to 0], 4	a. p=0.32<br>b. p=0.51	a. p=**0.03**<br>b. p=**0.04**<br>c. p=**0.03**
No. of HR- and pHR-HPV-types[b,c]	1 [1 to 3], 7	2 [1 to 3], 6	1 [1 to 2], 5	a. p=0.22<br>b. p=0.30	1 [1 to 2], 7	1 [1 to 3], 7	1.5 [1 to 2], 6	a. p=0.33<br>b. p=0.63	a. p=0.63<br>b. p=0.29<br>c. p=0.60
No. of LR-HPV-types[d] (in samples with and without HR/pHR-types)	1 [0 to 1], 3	1 [0 to 1], 3	1 [0 to 1], 3	a. p=**0.01**<br>b. p=**0.04**	1 [0 to 2], 5	1 [0 to 1], 5	1 [0 to 1], 4	a. p=1.00<br>b. p=1.00	a. p=0.46<br>b. p=0.72<br>c. p=0.50
No. of LR-HPV-types[d] (in samples without HR/pHR-types)	1 [1 to 2], 3	1 [1 to 1], 3	1 [1 to 1], 3	a. p=0.50<br>b. p=0.20	1 [1 to 1], 3	1 [1 to 1], 1	1 [1 to 2], 3	a. p=1.00<br>b. p=1.00	a. p=1.00<br>b. p=1.00<br>c. p=1.00
No. of HPV16/18/31/33/45/52/58[e]	1 [0 to 2], 4	2 [0 to 3], 4	1 [0 to 2], 4	a. p=0.15<br>b. p=0.78	2 [0 to 2], 5	2 [0 to 2], 4	2 [0 to 2], 5	a. p=0.93<br>b. p=0.72	a. p=0.42<br>b. p=0.82<br>c. p=0.22
**HPV-oncogene-mRNA detection**[f]<br>N (%) [95% CI]	72 (76.6%)<br>[66.7 to 84.7]	73 (76.8%)<br>[67.1 to 84.9]	73 (79.3%)<br>[69.6 to 87.1]	a. p=0.83<br>b. p=0.49	73 (76.8%)<br>[67.1 to 84.9]	71 (74.7%)<br>[64.8 to 83.1]	66 (73.3%)<br>[63.0 to 82.1]	a. p=0.82<br>b. p=1.00	a. p=1.00<br>b. p=0.87<br>c. p=0.39
**HPV-DNA loads**[g]<br>Median [IQR], n									
HPV16-DNA load	14.5 [0.6 to 136.8], n=35	9.4 [0.4 to 97.5], n=43	12.1 [1.2 to 65.1], n=35	a. p=0.79<br>b. p=**0.01**	9.4 [0.1 to 34.7], n=40	2.8 [0.4 to 41.0], n=41	4.6 [0.2 to 133.3], n=36	a. p=0.43<br>b. p=0.47	a. p=0.12<br>b. p=0.22<br>c. p=0.30
HPV16+18-DNA load	12.6 [0.8 to 101.0], n=10	3.0 [0.4 to 97.5], n=9	11.0 [1.1 to 64.3], n=10	a. p=0.34<br>b. p=0.91	9.2 [0.1 to 43.4], n=11	2.8 [0.3 to 35.2], n=8	2.8 [0.2 to 95.3], n=10	a. p=0.42<br>b. p=0.66	a. p=0.09<br>b. p=0.22<br>c. p=0.17
HR-HPV-DNA load[h]	35.6 [0.9 to 178.8], n=66	13.6 [1.4 to 137.8], n=69	17.1 [1.1 to 80.9], n=64	a. p=0.15<br>b. p=0.77	20.4 [0.5 to 112.4], n=69	6.2 [0.7 to 54.5], n=71	3.9 [0.4 to 101.8], n=65	a. p=**0.02**<br>b. p=0.85	a. p=0.28<br>b. p=0.18<br>c. p=0.12

For the legend, see legend of Supplementary Table S2.

Supplementary Table S4 Anal cytology results at baseline (bl), at post-treatment weeks 4 (4WFU) and 24 (24WFU) in the intention-to-treat (ITT) population.

**Time-point** / **Cytology**	**Total** n (%)	**ECA** n (%)	**TCA** n (%)	**p-value, ECA/ TCA**	**Difference** in % [95% CI], **ECA/TCA**	**p-value**, overall, **ECA/ TCA**	**p-value**, **ECA**, bl/FU, over time	**Difference** in % [95% CI], **ECA**, bl/FU, over time	**p-value**, overall, **ECA**, bl/FU, over time	**p-value**, **TCA**, bl/FU, over time	**Difference** in % [95% CI], **TCA**, bl/FU, over time	**p-value**, overall, **TCA**, bl/FU, over time	**p-value ECA**, 4W/24 WFU, over time	**Difference** in % [95% CI], **ECA**, 4W/24WFU, over time	**p-value**, overall, **ECA**, 4W/24 WFU, over time	**p-value**, **TCA**, 4W/24 WFU, over time	**Difference** in % [95% CI], **TCA**, 4W/24WFU, over time	**p-value**, overall, **TCA**, 4W/24WF U, over time
**Base-line**	n=233	n=115	n=118															
NILM	22 (9.4)	7 (6.1)	15 (12.7)	0.116	-6.6 [-14.1, 0.8]													
ASC-US	48 (20.6)	25 (21.7)	23 (19.5)	0.747	2.2 [-8.1, 12.6]													
ASC-H	0 (0.0)	0 (0.0)	0 (0.0)	1.000	0.0 [0.0, 0.0]	0.442												
LSIL	67 (28.8)	36 (31.3)	31 (26.3)	0.469	5.0 [-6.6, 16.6]													
HSIL	74 (31.8)	37 (32.2)	37 (31.4)	1.000	0.8 [-11.1, 12.8]													
missing	22 (9.4)	10 (8.7)	12 (10.2)	-	-	-												
**4WFU**	n=221	n=112	n=109															
NILM	25 (11.3)	6 (5.4)	19 (17.4)	**0.005**	**-12.1 [-20.3, -3.8]**		1.000	0.7 [-5.3, 6.8]		0.355	-4.7 [-14.0, 4.6]							
ASC-US	37 (16.7)	21 (18.8)	16 (14.7)	0.473	4.1 [-5.7, 13.9]		0.622	3.0 [-7.5, 13.4]		0.381	4.8 [-4.9, 14.6]							
ASC-H	1 (0.5)	0 (0.0)	1 (0.9)	0.493	-0.9 [-2.7, 0.9]	0.086	1.000	0.0 [0.0, 0.0]	0.816	0.480	-0.9 [-2.7, 0.9]	0.559						
LSIL	51 (23.1)	28 (25.0)	23 (21.1)	0.526	3.9 [-7.2, 15.0]		0.306	6.3 [-5.4, 18.0]		0.436	5.2 [-5.9, 16.2]							
HSIL	72 (32.6)	37 (33.0)	35 (32.1)	0.887	0.9 [-11.4, 13.3]		1.000	-0.9 [-13.1, 11.3]		1.000	-0.8 [-12.9, 11.4]							
missing	35 (15.8)	20 (17.9)	15 (13.8)	-	-	-	-	-	-	-	-	-						
**24WFU**	n=214	n=107	n=107															
NILM	27 (12.6)	11 (10.3)	16 (15.0)	0.411	-4.7 [-13.5, 4.2]		0.327	-4.2 [-11.4, 3.0]		0.700	-2.2 [-11.3, 6.8]		0.211	-4.9 [-12.0, 2.2]		0.713	2.5 [-7.3, 12.3]	
ASC-US	54 (25.2)	28 (26.2)	26 (24.3)	0.875	1.9 [-9.8, 13.5]		0.529	-4.4 [-15.7, 6.8]		0.421	-4.8 [-15.6, 6.0]		0.199	-7.4 [-18.4, 3.6]		0.087	-9.6 [-20.1, 0.9]	
ASC-H	0 (0.0)	0 (0.0)	0 (0.0)	1.000	0.0 [0.0, 0.0]	0.553	1.000	0.0 [0.0, 0.0]	0.698	1.000	0.0 [0.0, 0.0]	0.424	1.000	0.0 [0.0, 0.0]	0.523	1.000	0.9 [-0.9, 2.7]	0.277
LSIL	58 (27.1)	28 (26.2)	30 (28.0)	0.878	-1.9 [-13.8, 10.0]		0.459	5.1 [-6.7, 17.0]		0.767	-1.8 [-13.4, 9.9]		0.878	-1.2 [-12.7, 10.4]		0.270	-6.9 [-18.4, 4.5]	
HSIL	59 (27.6)	34 (31.8)	25 (23.4)	0.221	8.4 [-3.5, 20.3]		1.000	0.4 [-11.9, 12.7]		0.232	8.0 [-3.6, 19.6]		0.886	1.3 [-11.1, 13.7]		0.173	8.7 [-3.1, 20.6]	
missing	16 (7.5)	6 (5.6)	10 (9.3)	-	-	-	-	-	-	-	-	-	-	-	-	-	-	-

p-values < 0.05 are printed in bold. 4WFU, 4-weeks-follow-up (4 weeks after the end of treatment); 24WFU, 24-weeks-follow-up (24 weeks after the end of treatment); bl, baseline; CI, confidence interval; ECA, electrocautery; FU, follow-up; ITT, intention-to-treat; TCA, trichloroacetic acid.

In the TECAIN-study, cytology proved to be of limited value in assessing the therapeutic success of TCA and ECA treatment.
